# Cellular senescence and metabolic reprogramming model based on bulk/single-cell RNA sequencing reveals PTGER4 as a therapeutic target for ccRCC

**DOI:** 10.1186/s12885-024-12234-5

**Published:** 2024-04-11

**Authors:** Lijie Zhou, Youmiao Zeng, Yuanhao Liu, Kaixuan Du, Yongbo Luo, Yiheng Dai, Wenbang Pan, Lailai Zhang, Lei Zhang, Fengyan Tian, Chaohui Gu

**Affiliations:** 1 Department of Urology, First Affiliated Hospital of Zhengzhou University, 450052 Zhengzhou, Henan Province China; 2 Unit of Day Surgery Center, First Affiliated Hospital of Zhengzhou University, 450052 Zhengzhou, Henan Province China; 3 Department of Urology, Zhengzhou Key Laboratory for Molecular Biology of Urological Tumor Research, Henan Institute of Urology, First Affiliated Hospital of Zhengzhou University, 450052 Zhengzhou, Henan Province China; 4 Department of Thoracic Surgery, The First Affiliated Hospital of Zhengzhou University, 450052 Zhengzhou, Henan Province China; 5 Department of Pediatrics, The First Affiliated Hospital of Zhengzhou University, 450052 Zhengzhou, Henan Province China

**Keywords:** Renal cell carcinoma, Cellular senescence, Metabolism, PTGER4

## Abstract

Clear cell renal cell carcinoma (ccRCC) is the prevailing histological subtype of renal cell carcinoma and has unique metabolic reprogramming during its occurrence and development. Cell senescence is one of the newly identified tumor characteristics. However, there is a dearth of methodical and all-encompassing investigations regarding the correlation between the broad-ranging alterations in metabolic processes associated with aging and ccRCC. We utilized a range of analytical methodologies, such as protein‒protein interaction network analysis and least absolute shrinkage and selection operator (LASSO) regression analysis, to form and validate a risk score model known as the senescence-metabolism-related risk model (SeMRM). Our study demonstrated that SeMRM could more precisely predict the OS of ccRCC patients than the clinical prognostic markers in use. By utilizing two distinct datasets of ccRCC, ICGC-KIRC (the International Cancer Genome Consortium) and GSE29609, as well as a single-cell dataset (GSE156632) and real patient clinical information, and further confirmed the relationship between the senescence-metabolism-related risk score (SeMRS) and ccRCC patient progression. It is worth noting that patients who were classified into different subgroups based on the SeMRS exhibited notable variations in metabolic activity, immune microenvironment, immune cell type transformation, mutant landscape, and drug responsiveness. We also demonstrated that PTGER4, a key gene in SeMRM, regulated ccRCC cell proliferation, lipid levels and the cell cycle in vivo and in vitro. Together, the utilization of SeMRM has the potential to function as a dependable clinical characteristic to increase the accuracy of prognostic assessment for patients diagnosed with ccRCC, thereby facilitating the selection of suitable treatment strategies.

**Supplementary Information:**

The online version contains supplementary material available at 10.1186/s12885-024-12234-5.

## Introduction

Among urogenital malignancies, one of the most prevalent types is renal cell carcinoma (RCC) [1]. Breakthroughs have been made in surgical treatment, targeted therapies and combination therapy, including antiangiogenic therapy (such as the tyrosine kinase inhibitor sunitinib), mTOR inhibitors (such as rapamycin) and immunotherapy (such as the PD-1 inhibitor pembrolizumab) [2]. However, clinical outcomes, especially in patients with advanced RCC, still fall far short of expectations due to the development of resistance, the occurrence of adverse events, limited objective and durable responses and the high frequency of immune evasion [2–4]. The exploration of more effective methods for predicting RCC patient prognosis and guiding treatment decisions is an area that requires further investigation.

Metabolic reprogramming has developed as a hallmark of solid tumors, particularly RCC [5]. For renal cell carcinoma (RCC), the most prevalent form is clear cell renal cell carcinoma (ccRCC), comprising approximately 80% of all cases. This type of carcinoma is distinguished by mutations in the Von Hippel‒Lindau (VHL) allele, leading to alterations in tumor metabolism through the buildup of hypoxia-inducible factors (HIFs) [5]. Cancer cells of ccRCC are frequently accompanied by the Warburg-related dysfunction of the tricarboxylic acid (TCA) cycle and reprogramming of fatty acid (FA) metabolism to meet their energy and biosynthetic needs [5, 6]. Our previous research found that MLYCD-mediated fatty acid anabolism repressed ccRCC progression via regulation of endoplasmic reticulum stress and ferroptosis [7]. The reprogramming of metabolism in cancer cells is also associated with the development of the tumor microenvironment, which in turn promotes immune evasion [8]. Furthermore, cellular metabolism assumes a crucial function in the modulation of diverse signaling mechanisms implicated in cellular senescence. It is well known that, as a tumor suppressor gene, p53 induces several cell activities including apoptosis, cell cycle arrest, and senescence in cells and plays an essential role in controlling various metabolic pathways, which are responsible for maintaining cellular metabolic homeostasis and enabling cells to effectively respond to stressors [9, 10]. Cellular senescence has been recognized as a prominent feature of malignant tumors, signifying a condition of permanent cessation of growth in which cells remain metabolically active and release different proinflammatory and proteolytic substances as components of the secretory phenotype associated with senescence [11, 12]. Kawakami I et al. reported that the induction of cellular senescence in ccRCC can be achieved by specifically targeting the glutamine transporter SLC1A5 [13]. However, systematic and overall analysis of the landscape of metabolism and senescence in RCC remains lacking.

Our study identified the decisive genes related to cell senescence and metabolism and constructed and validated a senescence- and metabolism-related risk model for predicting ccRCC patient OS, employing Cox regression and LASSO regression analysis utilizing various expression matrices and single-cell sequencing data pertaining to ccRCC, which demonstrated a significant level of precision in predicting overall survival (OS) and guiding clinical treatment measures. We also demonstrated that PTGER4, a crucial gene in SeMRM, was found to exert regulatory control over cell proliferation, lipid metabolism, and cell cycle progression in both in vivo and in vitro models of ccRCC.

## Methods

### Data acquisition

The TCGA-KIRC database was obtained as a training dataset from UCSC Xena. Two independent ccRCC datasets, the ICGC KIRC (BGI, Nat Genet 2012) and GSE29609 cohorts, were downloaded as training sets from the cBioPortal and Gene Expression Omnibus (GEO) [14, 15]. The ccRCC single-cell sequencing data GSE156632 was acquired from the GEO database. The MRG and SRG datasets were acquired from MSigDB, a database that contains molecular characteristics information. The immune checkpoint data, on the other hand, were gathered from relevant literature sources [16].

### Differential gene expression analysis and visualization

We employed the “limma” R package (version 3.54.2) to conduct a screening of differentially expressed genes (DEGs). The criteria for identifying DEGs were set at a p value < 0.05 while the|FC| > 1.5 in absolute value [17]. To visually represent the DEGs, we utilized either volcano plots or heatmaps created using the R “ggplot2” package. Additionally, we employed Venn diagrams to illustrate the overlap between DEGs, MRGs, and SeRGs, with the aim of identifying SeMDEG.

### Gene ontology analysis

Performing Gene Ontology (GO) analysis with the R software package “clusterProfiler” (Version 4.6.2) to catch the potential meanings of the DEGs [18]. Statistical significance was determined based on adjusted p values < 0.05.

### Kyoto encyclopedia of genes and genomes analysis

Performing the KEGG analysis with the R software packages “clusterProfiler” and “enrichplot” were used to visualize. This analysis aimed to identify signaling pathways that may be linked to DEGs or SeMRM. The significance cutoff criteria were set at *p* < 0.05 and an FDR < 0.25 [18].

### Unsupervised clustering analysis

We used the R software package ConsensusClusterPlus for consistency clustering analysis [19]. The determination of the ideal quantity of clusters is accomplished through the utilization of the algorithm for clustering that maintains consistency. To ensure the clustering process stability and the reliability of the obtained results, the algorithm is repeated 1000 times.

### Construction of the PPI network

Employing the STRING online database to construct the SeMDEGs` PPI network. Then, the resulting web was brought in Cytoscape software for more analysis. The hierarchical arrangement of hub genes was established through the utilization of the CytoHubba plug-in. Subsequently, the top 100 genes were elected for advance analysis [20].

### Structure and verification of senescence-metabolism-related models for assessing aging-metabolism-related risk scores

In this study, performing the univariate Cox regression analysis to identify DEGs associated with OS using a p value < 0.05. Later, the LASSO analysis was performed. Genes exhibiting an HR and 95% CI below 1 were categorized as genes with a protective effect, while those with an HR and 95% CI greater than 1 were categorized as risk genes. The correlation between gene expression was assessed using the R package “corrplot”. Subsequently, LASSO analysis was conducted through the “glmnet” R package to mitigate the risk of overfitting recurrence characteristics and restrict the genetic variables considered in predicting overall survival. By integrating DEGs with multiple Cox analysis, the genes that were identified as significant through LASSO regression were subsequently assessed. The process of constructing the SeMRM model entails the allocation of weights to the estimated coefficients derived from the analysis of the Cox regression. The mathematical representation of the model is SeMRM = ∑ (bi × Expi), where bi represents the coefficient and Expi represents the normalized expression level of the model gene for each subscale. Based on the median SeMRM as the verge, all ccRCC samples were categorized into two subgroups: low SeMRS and high SeMRS [21].

To evaluate the discrepancy in recurrence rates observed within the two distinct risk score groups, Kaplan‒Meier analysis was employed. Furthermore, the “survivalROC” was utilized to generate the ROC curve, which enabled the assessment of the specificity and sensitivity of the SeMRM method making use of the AUC. Subsequently, to confirm the model’s prognostic meaning, independent of other factors, we conducted univariate and multivariate Cox regression analyses. Last, the efficiency of SeMRM was additional confirmed through another separate cohorts of ccRCC patients, ICGC-KIRC and GSE29609.

### Gene mutation analysis

From cBioPortal, we acquired genetic alterations, and the R “Maftools” package was employed to assess the quantity and characteristics of mutations within the two subgroups of SeMRM [22].

### Comprehensive analyses of molecular and immune infiltration characters in diverse senescence-metabolism-related risk score subcategories

Through TCGA-KIRC, we employed five online tools, including CIBERSORT and TIMER, to assess the immune infiltration characteristics of this cancer type. Subsequently, our study compared the immune cell profiles of the two SeMRM subgroups. Furthermore, using the “corrplot”, we conducted an external correlation analysis to examine the correlation between recurrence risk scores and checkpoints of immune. To evaluate the SeMRM`s prognostic meaning in response to immunotherapy, we utilized TIDE, an online tool, to anticipate how each patient will respond to inhibitors of immune checkpoints.

### Forecast of two TCGA-KIRC SeMRS Groups` drug sensitivity

Using the CTRP and GDSC databases, the R package “oncoPredict” was employed to forecast the reaction to various drugs used in chemotherapy. Our study conducted a comparative analysis of the variance in 50% inhibitory concentration (IC50) values within two SeMRS groups [23].

### Human samples

Following ethical guidelines, we obtained their consent after being informed from the patients and obtained the authorization from the First Affiliated Hospital of Zhengzhou University`s institutional research ethics committee. Subsequently, fresh renal cancer tissues were attained from the First Affiliated Hospital of Zhengzhou University, as were the corresponding adjacent normal tissues. These tissues were then stored at a temperature of -80 °C. Subsequently, we prepared paired sections of ccRCC and nearby renal tissues by embedding them in paraffin.

### Immunohistochemical staining assay

The IHC assay was conducted in accordance with previously established protocols (20). To maintain consistency in the study, the IHC tries were conducted through identical tissue chips [24]. The main antibodies comprised anti-ENO1 (1:2000, ab227978, Abcam), anti-NME2 (1:500, ab131329, Abcam), anti-CD44 (1:100, ab51037, Abcam), anti-ENO2 (1:500, ab79757, Abcam), anti-PTGER4 (1:250, 24895-1-AP, ProteinTech), anti-COL1A1 (1:5000, 67288-1-lg, ProteinTech) and anti-FGF1 (1:300, bs-0229R, Bioss). In our study, the expression of proteins in the tissue chips was assessed through the immunoreactive score (IRS) system. A score of 0–1 indicates negative staining, a score of 2–3 indicates mild staining, a score of 4–8 indicates moderate staining, and a score of 9–12 indicates significant positive staining on the IRS scale.

### Multiplex immunofluorescence staining assay

Through ImageJ software, the protein expression of the tissue chips was assessed by the corrected total cell fluorescence (CTCF) method. Acquiring the visualization and statistical analyses of data through R. Performing the comparison of continuous variables between various SeMRS subgroups through the independent t test, while using the chi-square test in the analyses of classification data. We employed the Wilcoxon test to compare the SeMRS scores among different TIDE immunotherapy response groups. The Pearson correlation coefficient was utilized to quantify the relationship between two continuous variables. To conduct the Univariate survival analysis by using the KM method and log rank test, while running the multivariate survival analysis using the Cox regression model. A p value below 0.05 was deemed the threshold to determine statistical significance.

### Cell culture and transfection

The RCC lines of human were obtained from the American Center for Type Culture Collection (LGC Standards, London, England). The RCC lines of human underwent routine monitoring to detect any potential presence of mycoplasma pollution. Culturing cells with Dulbecco’s modified Eagle’s medium (DMEM) (Thermo Fisher, Waltham, MA, USA) supplemented with 10% fetal bovine serum (FBS, Invitrogen, Carlsbad, CA, USA) and 1% penicillin‒streptomycin liquid (Gibco, Carlsbad, CA, US) at 37 °C with 5% carbon dioxide. The cells were grown until they reached 60–80% confluence and were then transfected with HitransG P virus infection reagent according to the manufacturer’s directions.

### Western blot

RIPA buffer (Solarbio, Beijing, China) accompanied with 1% PMSF was utilized for the extraction of total protein content from the cells. The quantification of protein was performed using the BCA Protein Assay Kit (Solarbio). 20 µg of protein was alienated using SDS‒PAGE gel equipped with the PAGE gel kit. The isolated proteins were then immediately transferred into the nitrocellulose membranes (Millipore, Danvers, MA, USA). To enhance the specificity of antibody binding, a blocking solution with a concentration of 1 was applied. During the incubation process, the primary antibody was applied to the nitrocellulose membrane, anti-PTGER4 (1:1000), while anti-GAPDH antibody (1:10000, ab9485, abcam) for loading control. The target bands were identified by the imaging system (Gene Company Limited, Hong Kong, China).

### Cell counting Kit-8 assay

Using the CCK8 assay (MedChem Express) to assess the ccRCC cell lines` proliferation activity. A cohort of 3000 cells was cultivated in 96-well plates containing 100 µL of DMEM within 10% FBS for a duration of 96 h. In each well, 10 µL of CCK8 was appended, and the cells were raised for 2 h. The DNM-9606 Microplate Reader, manufactured by Perlong in Beijing, China, was employed for measuring absorbance at a wavelength of 450 nm on a daily basis with a 24-hour interval.

### EdU assay

An EdU detection kit was used to assess the proliferation of cell. Cells were treated overnight at 3 × 10^4^ cells/well. Next, a concentration of 50 µmol/L of EdU was exposed to maturation at 37 °C for 6 h. Then, samples were fixed using paraformaldehyde for a period of 16 min and consequently permeabilized for a duration of 20 min using 0.5% Triton X-100. Treating cells with 100 µl 1× Apollo® reaction cocktail for a duration of 30 min, and the most important thing in this process was to avoid light at average temperature in a room. Finally, the nuclei were subjected to staining within DIPA 5 min and subsequently examined using fluorescence microscopy.

### Colony formation assay

Approximately 1000 ccRCC cells were placed on plates. The culture medium, consisting of DMEM supplemented with 10% FBS, was refreshed every 72 h. After a cultivation period of 14 days, the cells underwent a washing process using PBS followed by fixation with paraformaldehyde for a duration of 15 min. Next, PBS was used three times to rinse the portions. The crucial step in the process was to stain with 0.1% crystal violet for 30 min. After washing and then drying at room temperature, the clones were meticulously counted.

### Biochemical analysis

According to the manufacturer’s plan, the amounts of CHO, TG and FFA within the cell homogenate were analyzed by their respective detection kits.

### Staining of senescence-associated β-galactosidase (SA-β-Gal)

In compliance with the manufacturer’s instructions, the activity of SA-β-Gal was assessed utilizing a kit (Beyotime, C0602). Observing SA-β-Gal-positive cells using an optical microscope. The cell number was measured using the ImageJ program.

### In vivo tumor xenograft model

As per the statistical methods’ requirements, we conducted experiments by randomly selecting 5 mice from each group, which aligns with the approach followed in most articles. C57BL/6 mice (4–5 weeks of age, male) were acquired from Sibeifu Company (Beijing, China). 6 × 10^6^ ccRCC cells were separately infected with vectors and PTGER4. The cells were mixed with 50 µL of Matrigel (Corning) and 50 µL of PBS and injected under the skin on the back of mice. Conducting power analysis to determine the proper sample amount within our experimental study. All mice were randomly assigned to various sets. Starting from the 7th day, the size of the tumor was monitored and assessed every 3 days. Until the 40th day, the tumor was extracted, and its weight was recorded. At the end of the experiment, the mice were anesthetized with 1-1.5% isoflurane and then subjected to cervical dislocation. Then, we dissected the mice and calculated the size and weight of the subcutaneous tumors in the mice. In addition, remove the subcutaneous tumor, fix it in 4% paraformaldehyde for 24 h, and then dehydrate it. The experiment was conducted in accordance with the rules and regulations formulated by the Medical Ethics Committee of the First Affiliated Hospital of Zhengzhou University.

### Statistical analysis

We utilized R for all data visualization and statistical analysis. In the comparison of continuous variables, we used the independent t test. Employing chi-square test for the analysis of classification data. Utilizing the Wilcoxon test to assess the SeMRS within different TIDE immunotherapy response subgroups. Employing the Pearson correlation coefficient to evaluate the relationship between two continuous variables. A statistically significant result was described as having a p value < 0.05.

### Data availability

The article/supplementary material contains the novel contributions put forth in the study. Contact the corresponding author for additional inquiries.

## Results

### Detection and exploration of metabolic and senescence-associated genes in ccRCC

**Fig. 1:**
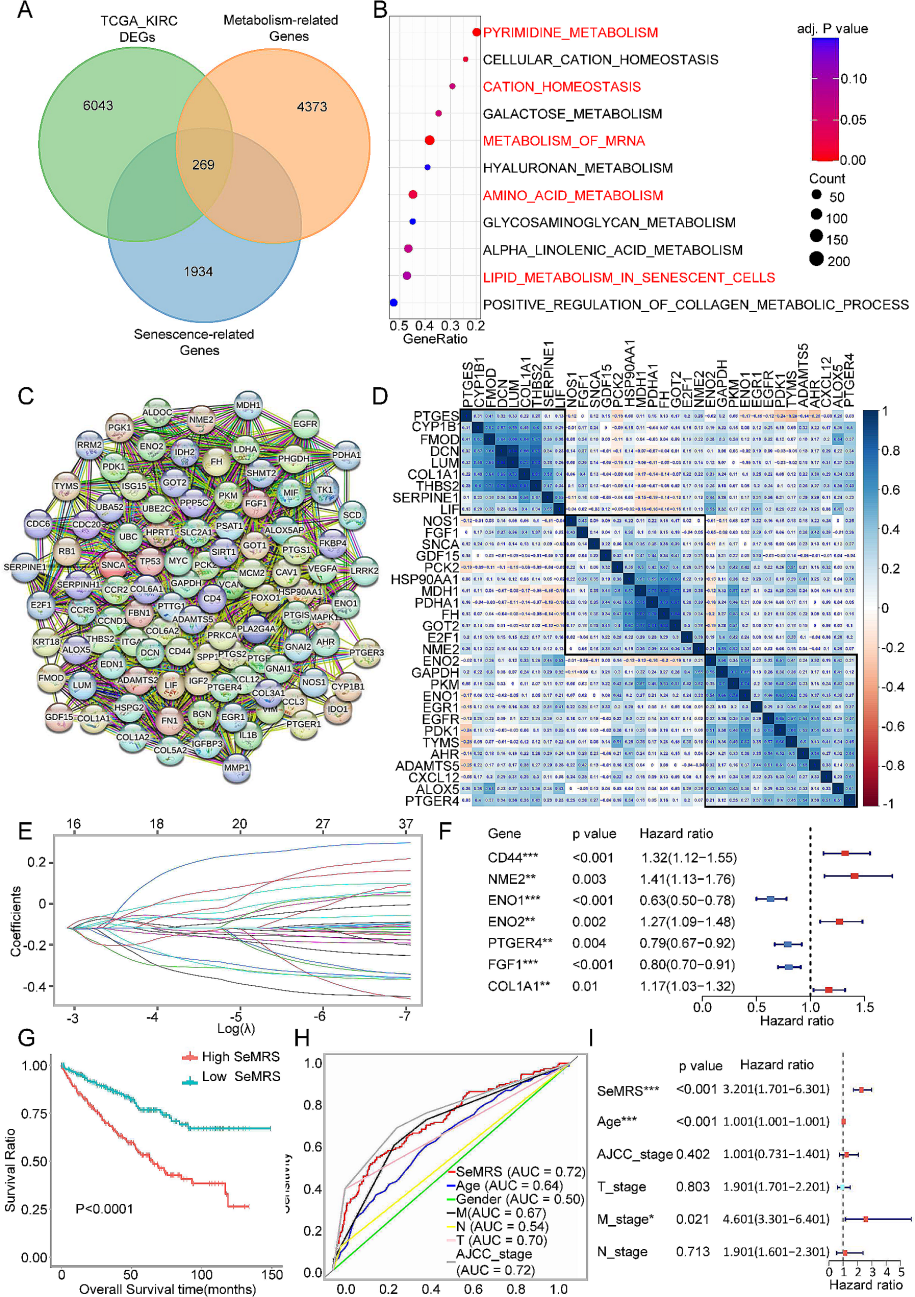
Identification, Investigating, Construction and analysis of differentially expressed genes (SeMDEG) related to aging metabolism in renal cell carcinoma (RCC). (**A**) Venn diagram of aging metabolism-related differential genes (SeMDEGs). *p* < 0.05,|FC| > 1.5. Bubble plots of the first 10 terms in (**B**) Kyoto Encyclopedia of Genes and Genomes (KEGG) mDEG enrichment analysis. After adjustment, *p* < 0. 01, *p* < 0. 05. (**C**) The protein‒protein interaction (PPI) network of the top 100 hub genes of these mDEGs. (**D**) Correlation of OS-related key mDEG. (**E**) Least absolute shrinkage and selection operator (LASSO) Cox regression of OS-related key senescence-metabolism-related differentially expressed genes (mDEG). (**F**) Multivariate Cox regression analysis was performed on 7 genes based on cross-validation and minimum partial likelihood bias to further demonstrate independent prognosis-related genes and obtain a gene index. (**G**) Kaplan‒Meier analysis of OS curves in TCGA renal cell carcinoma patients with low or high SeMRM subgroups. **(H)** ROC analysis showed that the predictive accuracy of SeMRM was better than that of other clinical features in the TCGA-KIRC cohort. **(I)** Multivariate Cox regression analysis of SeMRM and clinical features

To thoroughly investigate tumor metabolism at the genetic level in ccRCC, we conducted a systematic analysis by identifying the differentially expressed genes (mDEGs) associated with metabolism and senescence using the TCGA-KIRC database. Clear cell carcinoma of the kidney was compared with normal renal tissue, and 269 DEGs were recognized using a p value < 0.05 and the|FC| > 1.5 as a threshold to identify significance (Fig. 1A and Supplementary Fig. 1A). Through KEGG and GO enrichment analysis, the possible functional consequences associated with noteworthy genes were revealed, and the related pathway expression information of ccRCC metabolic panoramic changes was obtained. The following list presents the ten most significant GO terms associated with enrichment: BP includes extracellular matrix organization, acute inflammatory response, cell fate commitment, steroid metabolic process, carboxylic acid transport and lipid transport; CC includes collagen − containing extracellular matrix, transmembrane transporter complex, Golgi lumen, endoplasmic reticulum lumen and plasma lipoprotein particle; and MF encompasses various activities such as receptor ligand activity, extracellular matrix structural constituent, signaling receptor activator activity and G protein − coupled receptor binding (Supplementary Fig. 1B). The KEGG results showed that the DEGs were linked to various pathways in senescent cells, including pyrimidine metabolism, cation homeostasis, mRNA metabolism, amino acid metabolism, and lipid metabolism (Fig. 1B).

So as to study whether metabolic and senescence changes are related to the prognosis and survival of renal cell carcinoma, we conducted a consensus clustering analysis to categorize tumor tissues into different groups based on mDEG expression (Supplementary Fig. 1C-D). Two distinctive models were labeled: 189 cases for the first group and 303 cases in the second group (Supplementary Fig. 1E). Demonstrated notable distinctions between the two subtypes characterized by senescence and metabolism through survival analysis of Kaplan‒Meier (KM) using overall survival (OS) (Supplementary Fig. 1F). Substantially, the aforementioned findings suggest that the process of tumor senescence and the reprogramming of metabolism significantly help improve the prediction and overall survival of ccRCC.

### Screening differentially expressed senescence-metabolism-related key survival-related genes of renal cell carcinoma

First, to form a protein‒protein interaction (PPI) network, we utilized the STRING database with the aim of identifying the key genes among 269 DEGs. According to the score, the 100 most important genes are believed to have a greater significance in the survival and prognosis of ccRCC (Fig. 1C). Next, an analysis of the 100 central genes was conducted using KEGG and GO methodologies. The KEGG disclosed that the pointed mDEGs were found to be augmented in metabolic pathways, pathways in cancer, carbon metabolism, cellular senescence and the cell cycle, which were associated with ccRCC proliferation and development. The aggregation of the ten most enriched GO terms included unsaturated fatty acid metabolic process, response to peptide, and Golgi lumen. The results displayed in Supplementary Fig. 2A-B indicate that key mDEGs have a major impact on metabolism, senescence, and renal cancer progression. Next, to identify OS-related DEGs, we operated univariate Cox analysis via the top 100 hub genes. According to the data presented in Supplementary Fig. 2C, a statistical analysis using a p value threshold of less than 0.05 revealed that of the top 100 genes, 32 genes were found to have a significant association with overall survival (OS). Among these genes, 14 exhibited risk factors with hazard ratios (HR) and 95% confidence intervals (CI) > 1, indicating an increased risk. Conversely, 18 genes demonstrated protective effects. As depicted, the majority of the crucial mDEGs related to survival are interrelated, indicating that the alteration of senescence and metabolism in ccRCC involves a comprehensive modification rather than a singular genetic mutation (Fig. 1D).

### Manufacture of a SeMRM for predicting the OS of patients with ccRCC

To create a thorough and efficient senescence-metabolism-related risk prediction model (SeMRM), we conducted LASSO-Cox regression analysis on key mDEG associated with OS. Following cross-validation, we identified seven genes (CD44, ENO1, ENO2, FGF1, COL1A1, PTGER4, and NME2) that exhibited minimal partial likelihood bias and were therefore emphasized (Fig. 1E and Supplementary Fig. 2D). Then, to further illustrate genes that are independently associated with prognosis and to generate a gene index, we performed multivariate Cox analysis. As depicted, CD44, NME2, ENO2 and COL1A1 were independent risk factors, and ENO1, PTGER4 and FGF1 were independent protective factors (Fig. 1F). The prognostic SeMRM was developed using the subsequent equation: SeMRS = 0. 28 * CD44 + 0. 34 * NME2 + 0. 24 * ENO2 + 0. 16 * COL1A1-0. 46 * ENO1-0. 24 * PTGER4-0. 22 * FGF1 expression, where SeMRS represents the senescence-metabolism-related risk score.

Next, the SeMRS was computed based on the established model. Subsequently, 492 patients diagnosed with ccRCC were categorized into two subgroups, using the median SeMRS as the dividing point (Supplementary Fig. 2E). As depicted in Fig. 1G, a notable disparity in the occurrence of unfavorable prognosis between the low-SeMRS subgroup and the high-SeMRS subgroup was disclosed. This suggests that higher SeMRS is coupled with poorer OS for patients. As shown from the ROC curve, SeMRM could be used as a predictive clinical characteristic. The AUC values for overall survival (OS) at 1, 3, and 5 years were 0.747, 0.713, and 0.715, respectively. These values suggest that both high sensitivity and specificity were demonstrated by SeMRM (Supplementary Fig. 2F). Furthermore, from the ROC curve, SeMRM had a higher accurateness in assuming overall survival than other clinical elements, containing TNM stage, gender, and age (Fig. 1H). We also investigated the relationship among SeMRS, clinical characteristics, and the expression of mDEG from TCGA-KIRC. The heatmap indicated that CD44, NME2, ENO2, and COL1A1 were higher in the high SeMRS group, whereas ENO1, PTGER4, and FGF1 showed the opposite trend (Supplementary Fig. 3A). Additionally, we discovered that SeMRS exhibited a positive relationship between TNM stage and clinical stage. However, no correlation was observed with age (Supplementary Fig. 3B). Furthermore, the OS of ccRCC was found to be strongly associated with TNM stage, AJCC stage, age, and SeMRS based on the findings of the analysis of univariate Cox (Supplementary Fig. 2G). From multivariate Cox analysis, the SeMRS` p values and age (*p* < 0.001) were significantly lower than those for the other clinical features (Fig. 1I). However, the reliability of age as a prognostic indicator was found to be low. The above findings indicate that SeMRS could be the most crucial prognostic indicator for ccRCC independently. Hence, these results indicate that the newly introduced aspect of SeMRM could serve as a more accurate prognostic indicator for ccRCC patient OS.

### Confirmation of the predictive significance of the senescence-metabolism-related risk model in two separate ccRCC units and real-world research

**Fig. 2:**
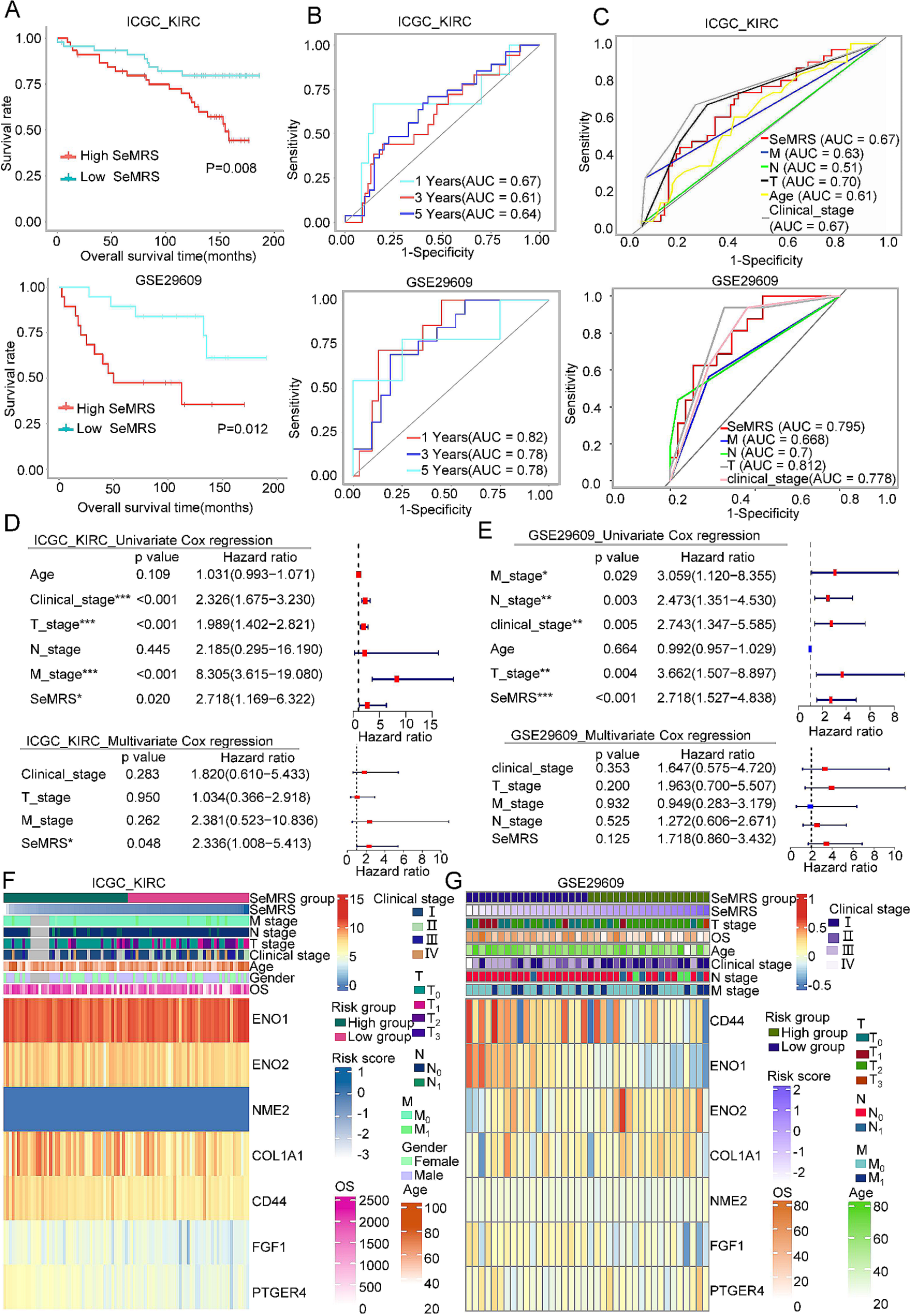
Prognostic value of the senescence-metabolism-related risk model (SeMRM) in two independent renal cell carcinoma (ccRCC) cohorts. (**A**) Kaplan‒Meier analysis of the overall survival (OS) curve of patients with low or high senescence-metabolism-related risk score (SeMRM) subgroups from two independent validation cohorts (International Cancer Genome Consortium (ICGC) KIRC and GSE29609). (**B**) Receiver operating characteristic (ROC) curve for predicting 1-year, 3-year and 5-year OS in the ICGC KIRC and GSE29609 cohorts. (**C**) ROC analysis showed that in the ICGC KIRC and GSE29609 cohorts, the predictive accuracy of SeMRM in OS was better than that of other clinical features. Univariate and multivariate Cox regression analysis of SeMRM and clinical features in the (**D, E**) ICGC KIRC and GSE29609 cohorts. (**F, G**) Immunohistochemistry (IHC) staining was used to detect the expression of key metabolism-related differentially expressed genes (mDEGs) (NME2, CD44, COL1A1, ENO2, ENO1, FGF1, and PTGER4) in ccRCC tissue arrays from 61 normal tissues and 153 tumor tissues. A representative image is shown. Statistical analysis of the IHC staining immunoreactivity score (IRS). *p < 0. 05; **p < 0. 01; ***p < 0. 001

Confirming the predictive significance of SeMRM, we examined a separate KIRC database that contained information on the overall survival (OS) of ccRCC patients. We identified two relevant datasets in this database: the International Cancer Genome Consortium (ICGC) KIRC cohort and GSE29609. As previously stated, SeMRS was determined using the senescence-metabolism-related risk model (SeMRM) equation. Based on SeMRS, the samples were categorized into either high-SeMRS or low-SeMRS subgroups (Supplementary Fig. 3C). In line with the findings derived from the TCGA-KIRC database, individuals exhibiting elevated SeMRS levels experienced reduced overall survival duration and exhibited a more unfavorable prognosis (Fig. 2A). The 1-year, 3-year, and 5-year of AUCs for the predictions in ICGC KIRC data were 0.67, 0.61, and 0.64, respectively. Similarly, the AUC values of GSE29609 were 0.82, 0.78, and 0.78. These discoveries recommend that the use of SeMRM as a clinical feature may have significant potential in accurately and reliably predicting overall survival in patients with ccRCC (Fig. 2B). Furthermore, the ROC curves presented in Fig. 2C demonstrate that the predictive accuracy of SeMRM surpasses that of other clinical features in the ICGC KIRC and GSE29609 cohorts, with the exception of T stage, where it performs slightly less favorably. We also conducted an analysis to examine the correlation between overall survival (OS), clinical characteristics, and the expression of key mDEGs in these two databases. (Fig. 2F-G). The findings from the ICGC-KIRC and GSE29609 cohorts align with those from the TCGA-KIRC cohort. In the ICGC-KIRC set, the univariate Cox analysis revealed a noteworthy connection among OS, clinical stage, T stage, M stage, and SeMRS. However, only SeMRS remained meaningfully connected with OS in the analysis of multivariate Cox. Consequently, SeMRS could work as a prognostic indicator for ccRCC independently (Fig. 2D). A significant relationship between clinical stage, TNM stage, and SeMRS with OS in the GSE29609 set was showed by the analysis of univariate Cox. However, there was a weak correlation between these factors and OS through multivariate Cox analysis (Fig. 2E). This discrepancy may be ascribed to the occurrence of data heterogeneity resulting from the relatively small sample size. Therefore, these data suggest that SeMRM is the best independent predictor of OS in two separate ccRCC cohorts.

### Confirmation of the reliability of the senescence-metabolism-related risk model by single-cell data combined with ccRCC patient histochemistry chip data

**Fig. 3:**
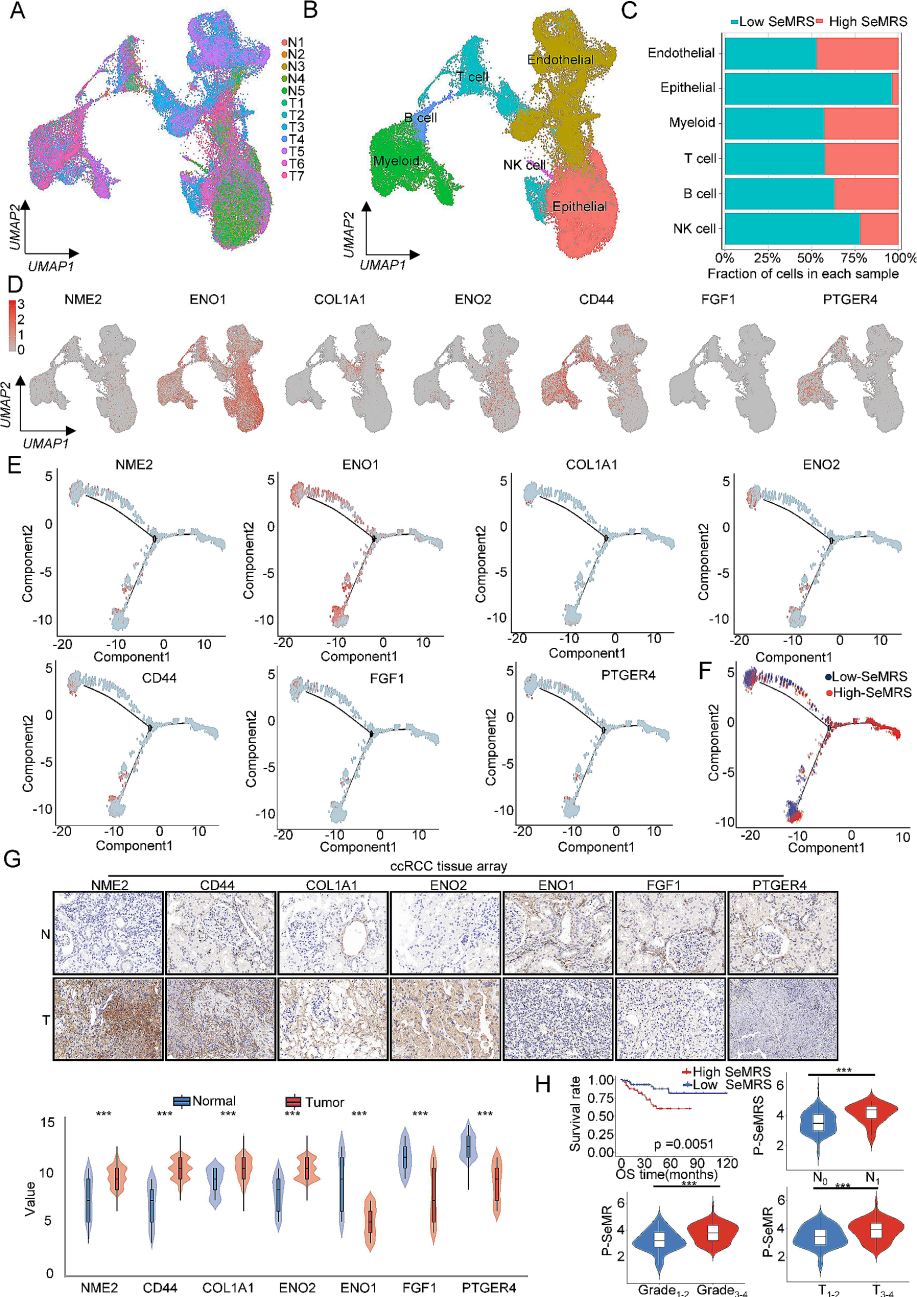
Prognostic value of the senescence-metabolism-related risk model (SeMRM) in single-cell data of renal cell carcinoma (ccRCC) and real-world studies. (**A-B**) UMAP maps of all single cells. (**A**) Each color encodes 12 sample sources. (**B**) Each color encodes 6 major cell types. (**C**) The proportion of cells derived from 5 nonmalignant and 7 tumor samples according to the SeMRM classification. (**D**) The expression of seven key mDEGs in the whole umap. (**E**) Expression and distribution of seven key mDEGs in the differentiation trajectory of ccRCC epithelial cells. (**F**) The differentiation trajectory of ccRCC epithelial cells with SeMRM as the classification standard. (**G**) Immunohistochemical (IHC) staining was performed using ccRCC tissue arrays from 61 normal tissues and 153 tumor tissues to detect the expression of key metabolism-related differentially expressed genes (mDEG) (NME2, CD44, COL1A1, ENO2, ENO1, FGF1, and PTGER4). A representative image is shown. Statistical analysis of the IHC staining immunoreactivity score (IRS). (**H**)According to the survival data of chip patients, a survival analysis diagram of high and low SeMRM patients was drawn. *p < 0. 05; **p < 0. 01; ***p < 0. 001

Subsequently, we divided the single-cell sequencing data of GSE156632 into 12 different categories (Fig. 3A and Supplementary Fig. 3D) according to the sample source and divided them into two different categories according to the classification criteria of cancer and adjacent cancer (Supplementary Fig. 3E). Cluster analysis was performed using the genetic data from the marker genes as described in the relevant articles in the database (the marker genes of some cell groups came from the cellmaker online tool). The sequencing data of 12 samples were divided into six categories: myeloid, NK cells, endothelial, epithelial, T cells and B cells (Fig. 3B). Subsequently, the SeMRS calculation formula was applied to the single-cell sequencing data, resulting in the division of the data into a high-SeMRS subgroup and a low-SeMRS subgroup using the median value. Subsequently, a histogram was generated to illustrate the proportion of high-SeMRS and low-SeMRS cells within each group (Fig. 3C). The findings indicated that there were notable variations in the proportions of high- and low-risk epithelial cells and NK cells, and an uneven proportion of cell groups appeared according to different classification criteria (Supplementary Fig. 3F). The key mDEGs also showed differential expression in different cell groups (Fig. 3D). Through cell communication analysis of the data, it was found that there was a strong intercellular communication connection between epithelial cells, endothelial cells, myeloid cells, T cells and B cells (Supplementary Fig. 4A-B). Subsequently, we grouped the data according to SeMRS and performed GO and KEGG analyses. The KEGG results were mostly enriched in cellular senescence, fatty acid metabolism, pyruvate metabolism and the citrate cycle (TCA cycle). The following are the top 10 GO terms for enrichment: BP includes fatty acid oxidation, negative regulation of immune system process, malnutritional system development, aging and T-cell mediated cytotoxicity; CC includes ER to Golgi transport vesicle membrane, low-density lipoprotein particle and protein-lipid complex. The MF results included fatty acid binding and T-cell receptor binding (Supplementary Fig. 4C-D). The previous analysis of the cell population histogram revealed notable disparities in the distribution of high and low SeMRS groups among epithelial cells. We performed a quasitime series analysis of the epithelial cell population and found that key mDEGs showed significant differences in expression at different developmental stages (Fig. 3E and Supplementary Fig. 4E). According to the classification of high and low SeMRS, there were also obvious distribution differences (Fig. 3F).

Next, we utilize the dataset gained from the First Affiliated Hospital of Zhengzhou University to investigate the practical significance of SeMRM. The protein expression of SeMRM was measured using immunohistochemistry (IHC), and the protein level was determined through the immune response score of OS-related mDEG (NME2, CD44, COL1A1, ENO2, ENO1, FGF1, PTGER4). As depicted, the protein expression of NME2, CD44, COL1A1, and ENO2 was markedly amplified in ccRCC tissues in comparison to normal renal tissues (Fig. 3G). Conversely, the expression levels of ENO1, FGF1, and PTGER4 exhibited contrasting results. Additional examination revealed that there was a positive relationship between pSeMRS and T stage, N stage, and clinical grade. Furthermore, with the survival data gained from the specimens collected, high-SeMRS patients with ccRCC exhibited an unfavorable prognosis (Fig. 3H). To summarize, these results indicate that SeMRS could be a potentially valuable predictive characteristic for patients with ccRCC, offering a high level of dependability and precision.

### The biological process and metabolic changes of the senescence-metabolism-related risk score in ccRCC

**Fig. 4:**
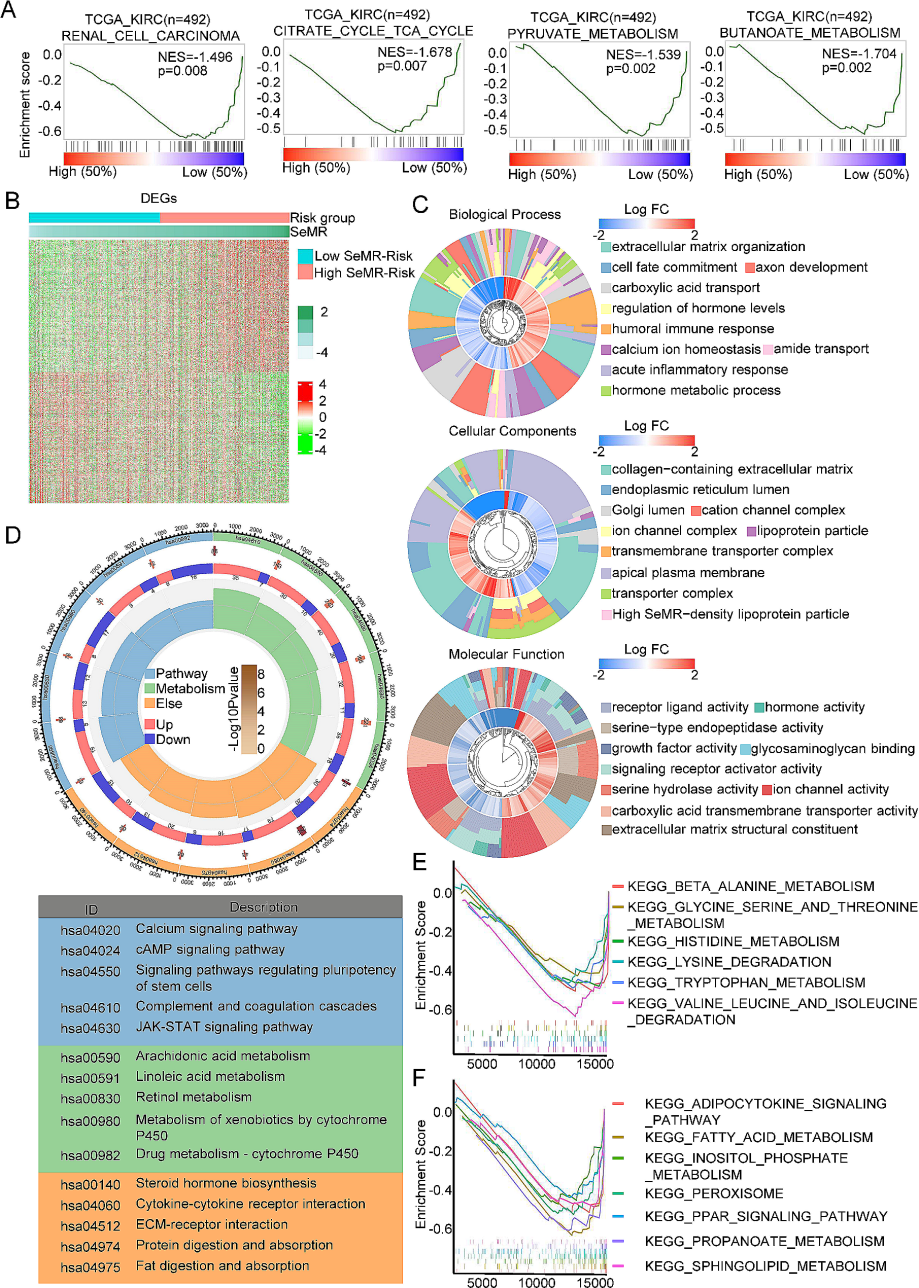
The molecular function and mechanism of the senescence-metabolism-related risk model (SeMRM) in clear cell renal cell carcinoma (ccRCC). (**A**) Senescence metabolism-related risk score (SeMRM) and gene set enrichment analysis (GSEA) of the renal cancer progression signaling pathway. *P* < 0. 05 and false discovery rate (FDR) < 0. 05 were considered significant. (**B**) Comparison of the heatmaps of differentially expressed genes (DEGs) between the high-SeMRM group and the low-SeMRM group. *P* < 0. 05,| FC| > 1. 5. (**C**) Gene Ontology (GO) analysis was used to explore the molecular functions and biological processes of these DEGs. (**D**) Kyoto Encyclopedia of Genes and Genomes (KEGG) analysis revealed the main pathways involved in these DEGs. (**E**) Gene sets related to amino acid metabolism in high or low SeMRM subgroups (*p* < 0. 05, FDR < 0. 25). (**F**) Gene sets related to lipid metabolism in high or low SeMRM subgroups (*p* < 0. 05, FDR < 0. 25)

Initially, we conducted gene set enrichment analysis to forecast alterations between the high-SeMRS subgroup and the low-SeMRS subgroup within the TCGA-KIRC cohort. Our results showed that the low-SeMRS was enriched in renal cell carcinoma, citric acid metabolism, pyruvate metabolism and butyric acid metabolism (Fig. 4A), indicating that there were notable variations in tumor growth and metabolism among the different subgroups of SeMRS. After performing gene expression analysis, 391 genes were labeled through a comparative analysis between the high-SeMRS and the low-SeMRS (Fig. 4B). Additional research have found that DEGs are mainly enriched in a series of metabolic activities, material transport and cell growth and development (Fig. 4C). KEGG analysis revealed a strong association between the DEGs and various metabolic pathways, including the cAMP signaling pathway, arachidonic acid metabolism, steroid hormone biosynthesis, protein digestion and absorption, and fat digestion and absorption (Fig. 4D). As showed in Fig. 4E, the metabolic pathway characteristics obtained by KEGG analysis, specifically the amino acid metabolic pathway and lipid metabolism. The findings indicated that the group with low SeMRS exhibited a greater prevalence of amino acid metabolism and degradation, as well as fatty acid metabolism, in comparison to the high SeMRS group. Furthermore, GO and KEGG indicated that DEGs were also associated with receptor activity and body immunity, which indicated that SeMRM was related to cellular immunity, and subcategories had dissimilar immune microenvironments (Fig. 4C-D).

### Immune and mutation features of subgroups with different senescence-metabolism-related risk scores

**Fig. 5:**
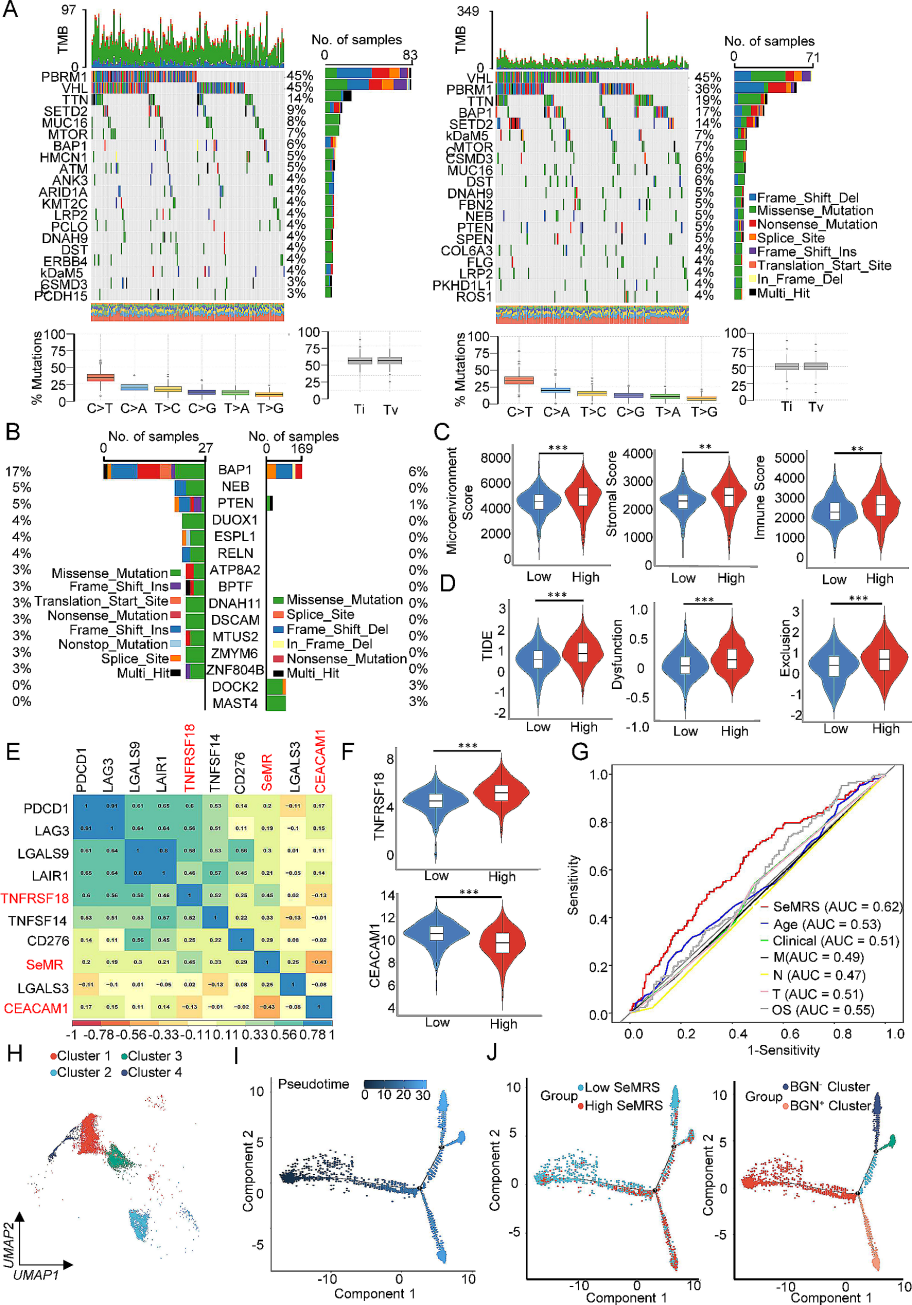
Immune and mutation characteristics of subgroups with different metabolic-related risk scores. (**A**) The top 20 genes with higher mutation rates in the high or low SeMRM groups and related mutation categories. (**B**) The 15 genes with the most obvious difference in mutation between the high and low SeMRM groups. (**C-D**) Immune microenvironment and immunotherapy-related scores in the high and low SeMRM groups. (**E**) Correlation between SeMRM and classical immune checkpoints. (**F**) The expression of TNFRSF18 and CEACAM1 in the high and low SeMRM subgroups. (**G**) Receiver operating characteristic (ROC) analysis showed that the predictive accuracy of the metabolic-related risk model (SeMRM) for the response to immunotherapy was slightly higher than that of other clinical features in the TCGA-KIRC cohort. (**H**) T-cell subset umap; different colors represent different subsets. (**I**) Differentiation trajectory of T cells in ccRCC; each color is encoded as false time (top) and cluster (bottom). (**J**) High or low SeMRM subtypes and marker gene high or low expression subtype T-cell subset pseudotime series analysis. **p < 0. 01; ***p < 0. 001

Then, we analyzed the gene mutations to delve deeper into the genetic variations in the SeMRS subgroup. The most prevalent form of mutation observed was missense mutation (Supplementary Fig. 5A), and the high-SeMRS exhibited a faintly greater number of mutations equated with the low-SeMRS. As Fig. 5A depicts, the genes exhibiting the most pronounced mutation rates within the SeMRS subgroup. In the high-SeMRS subtype, the mutation rates of DOCK2 and MAST4 were found to be higher than the low-SeMRS subtype, while these genes` mutation rates including BAP1, NEB, PTEN, DUOX1, ESPL1, RELN, ATP8A2, BPTF, DNAH11, DSCAM, MTUS2, ZMYM6 and ZNF804B were lower than the low-SeMRS subgroup (Fig. 5B); pBRM1, VHL and TTN showed high mutation rates in both subtypes. The immediate surroundings of tumor cells, known as the tumor microenvironment (TME), consist of immune cells, blood vessels located in the periphery, fibroblasts, the extracellular matrix and several signaling molecules. Firstly, we investigated the correlation between SeMRS and the TME. SeMRS exhibited a positive correlation using the TME score, whereby the high-SeMRS demonstrated superior levels of immune cell infiltration and matrix score compared to the low-SeMRS (Fig. 5C). Subsequently, we estimated the connection between SeMRS and the potential ability of tumor immune escape. Our research indicated a positive relationship between SeMRS and tumor immune escape. The high-SeMRS group exhibited a greater immune escape ability compared to the low-SeMRS (Fig. 5D). We conducted a comprehensive analysis of established immune checkpoints and curated a collection of these genes. Subsequently, we utilized these genes to delineate the functions related to the immune system and molecules that distinguish different subgroups of SeMRS. Our study revealed a strong relationship between the expression of nine immune checkpoint molecules and SeMRS (*r* > 0.19), including TNFRSF18 and CEACAM1 (Fig. 5E). It is noteworthy to mention that there was a noteworthy increase in TNFRSF18 expression in samples with high SeMRS, and CEACAM1 expression was meaningly decreased in low-SeMRS samples (Fig. 5F), indicating that immunotherapy has the potential to offer enhanced efficacy as a treatment option for these patients. In the assessment of immunotherapy response prediction, the AUC value of SeMRM surpasses other clinical features (Fig. 5G), suggesting that SeMRS, as a novel feature, holds promise as a prospective indicator for prognosticating the reaction to immunotherapy in individuals diagnosed with ccRCC. To acquire a deeper valuing of the influence of SeMRM from the immune milieu of people diagnosed with ccRCC, we performed subgroup cluster analysis (Fig. 5H) and pseudotime series analysis (Fig. 5I and Supplementary Fig. 5B) on the T-cell subsets in the previous single-cell data and classified the pseudotime series results according to the SeMRM score. The distribution of high and low SeMRM in different T-cell subsets was significantly different. Therefore, we analyzed the differences between these two subgroups and found that the BGN gene was expressed at low levels in T-cell subsets characterized by low SeMRS. The expression of the BGN gene in T-cell subsets with high SeMRM subgroup aggregation was also higher, so we believe that BGN is proportional to the SeMRM level and can be used as a specific indicator to predict the T-cell SeMRS level (Fig. 5J and Supplementary Fig. 3F).

### Drug sensitivity of renal cell carcinoma patients in different SeMRS subgroups

**Fig. 6:**
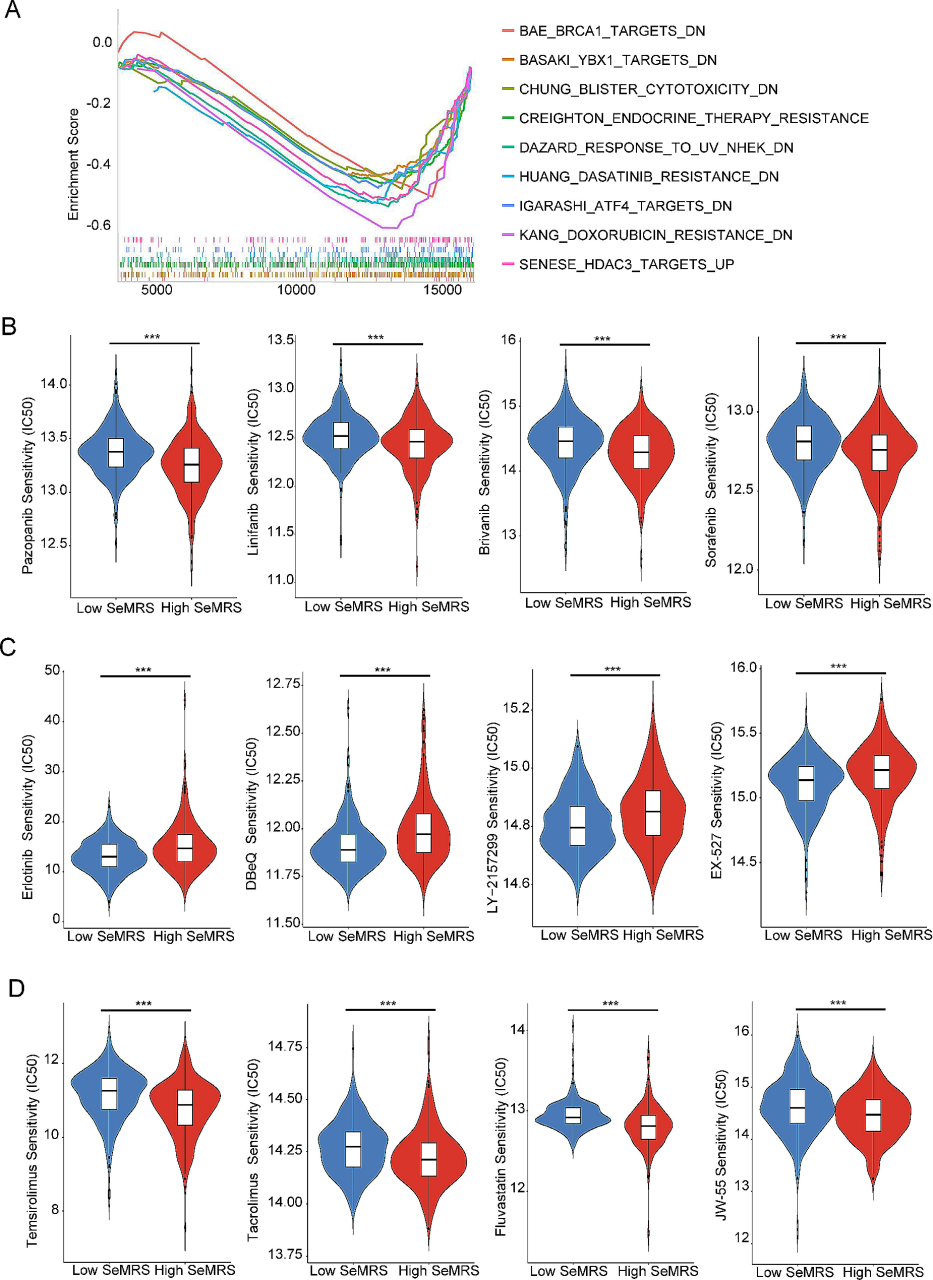
Sensitivity of clear cell renal cell carcinoma (ccRCC) patients with different senescence-metabolism-related risk score (SeMRM) subgroups to drugs. (**A**) Gene set enrichment analysis (GSEA) showed that drug metabolism-related gene sets were enriched in high or low SeMRM subgroups (*p* < 0. 05, false discovery rate (FDR) < 0. 25). (**B**) Sensitivity estimation of current clinically preferred drugs for patients with advanced renal cell carcinoma with high and low SeMRM risk. (**C, D**) to predict the sensitivity of potential drugs to advanced renal cell carcinoma in SeMRM high-risk and low-risk patients

Since GO and KEGG enrichment analysis (Fig. 4C-D) showed that SeMRS is related to hormone synthesis and metabolic pathways, chemotherapy and targeted drug therapy have always been an important part of the management of advanced RCC, we assume that patients belonging to distinct SeMRS groups have diverse sensitivities to drugs. We conducted a thorough examination of the hormone synthesis and metabolism pathways that were identified through KEGG enrichment analysis of the SeMRS subgroups. In our study, the pathways associated with reduced resistance to most chemotherapeutic drugs were associated with the low SeMRS group (Fig. 6A). Next, we assessed the responsiveness of the high-SeMRS and the low-SeMRS to typical anticancer medications to determine appropriate treatment choices for people with ccRCC. The low-SeMRS subgroup of patients with advanced renal cell carcinoma showed greater responsiveness to initial chemotherapy agents such as pazopanib and sorafenib in terms of standard drug selection and showed superior sensitivity to first-line medicines such as linifanib and brivanib for liver cancer (Fig. 6B). Regarding the recommended drugs, the high-SeMRS subpopulation showed high sensitivity to EGFR tyrosine kinase inhibitors (such as erlotinib), ATP-competitive p97 inhibitors (such as DbeQ), TGFβ receptor type I kinase inhibitors (such as LY-2,157,299) and SirT1 inhibitors (such as EX-527) (Fig. 6C). The low-SeMRS subgroup showed higher sensitivity to mTOR inhibitors (such as temsirolimus), calcineurin inhibitors (such as tacrolimus), competitive HMG-COA reductase inhibitors (such as fluvastatin) and selective β-catenin inhibitors (such as JW-55) (Fig. 6D).

### PTGER4 repressed cell senescence and lipid accumulation in RCC cells

**Fig. 7:**
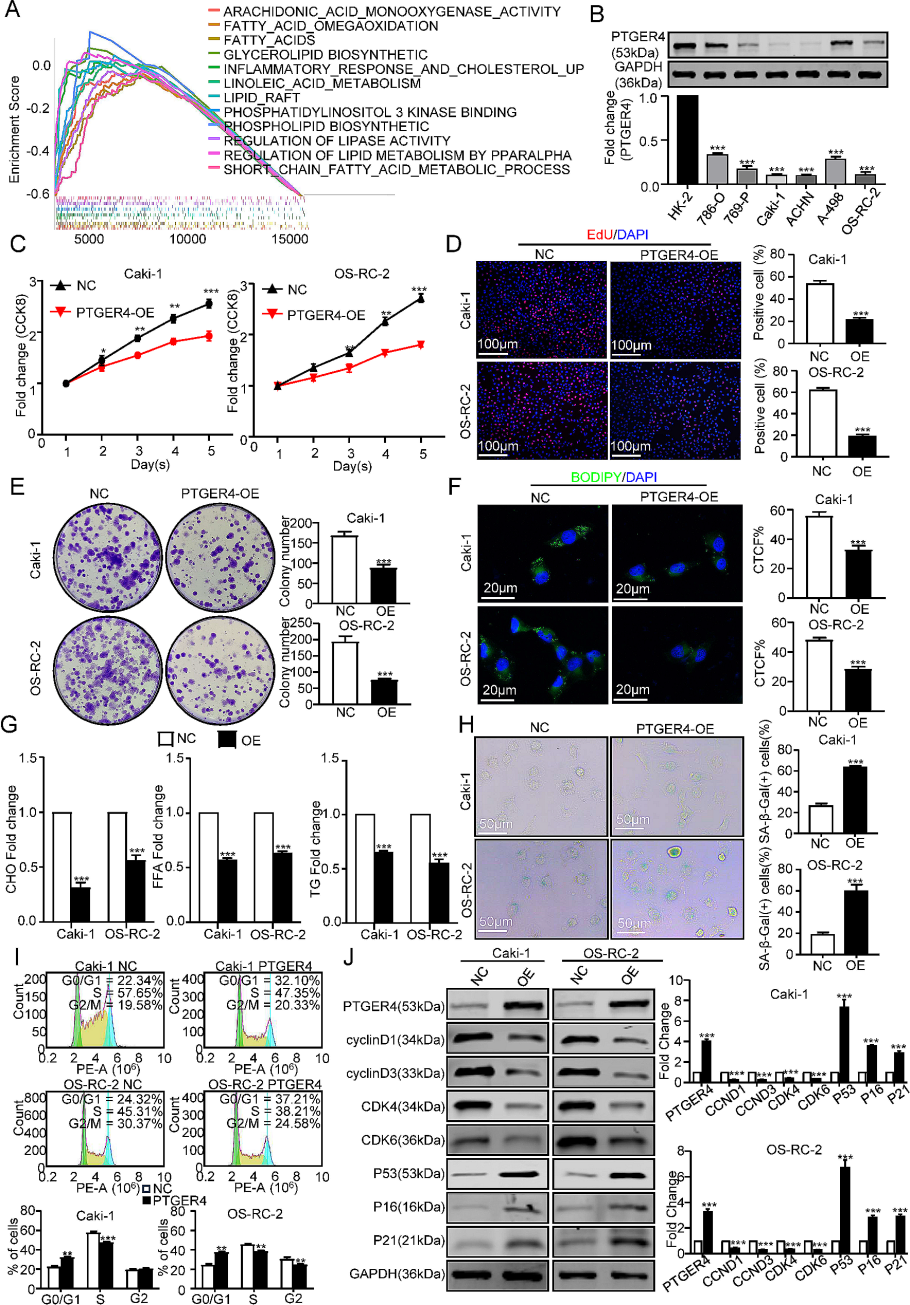
PTGER4 repressed cell senescence and lipid accumulation in RCC cells. (**A**) Gene set enrichment analysis (GSEA) showed that the differential analysis of high or low PTGER4 subgroups was mainly enriched in metabolic-related pathways. (**B**) Protein expression levels of PTGER4 in normal renal cells and renal cancer cells. (**C**) A CCK-8 assay was performed to detect RCC cell proliferation. NC is a blank vector control overexpressing PTGER4. The chart shows the mean ± SEM (*n* = 3 for each group). (**D**) EdU showed the proliferation ability of Caki-1 and OS-RC-2 cells after overexpression of PTGER4. There was no paired t test. (**E**) The proliferation ability of Caki-1 and OS-RC-2 cells after PTGER4 overexpression is shown. No paired t test, analysis of variance. (**F**) BODIPY staining showed the content of neutral fatty acids in Caki-1 and OS-RC-2 cells after PTGER4 overexpression. No paired T Test. (**G**) The content of CHO, FFAs and TG in Caki-1 and OS-RC-2 cells after PTGER4 overexpression (**H**) showed that the content of β-galactosidase in Caki-1 and OS-RC-2 cells after PTGER4 overexpression (**I**) showed cell cycle changes after PTGER4 overexpression. (**J**) Changes in the cell cycle and senescence-related protein expression after PTGER4 overexpression

Through the above research, we observed that SeMRS is faithfully related to metabolism. Therefore, we chose PTGER4, a gene closely associated with metabolism in SeMRM, to study the effect of its expression on renal cancer cell proliferation, invasion and migration in vitro and in vivo. Based on PTGER4 expression, we separated the TCGA-KIRC cohort into subgroups based on high and low levels of PTGER4. The KEGG cluster analysis revealed that the majority of the analysis findings in the two subgroups were enriched together in pathways related to metabolism, especially lipid metabolism (Fig. 7A), which verified our idea of selecting it as a representative gene. Subsequently, we collected normal kidney cells (HK-2) and various types of kidney cancer cell lines for protein analysis using western blot experiments. The findings indicated that PTGER4 exhibited low expression levels in cell lines of renal cell carcinoma (Fig. 7B). Subsequently, we selected Caki-1 and OS-RC-2 with moderately low expression of PTGER4 as representative RCC cells in our follow-up study. The lentiviral vector was utilized to facilitate the overexpression of the aforementioned cells, thereby augmenting PTGER4 expression. Subsequent experiments showed that the increase in PTGER4 suppressed the proliferation of renal cancer cell lines (Fig. 7C-E), and similar results were observed in both cell types. To study whether the difference in PTGER4 expression can affect the lipid content of renal cancer cell lines, we performed BODIPY staining on the cells in both the experimental unit and the control unit. Increased PTGER4 reduced the content of neutral lipid droplets in renal cancer cells (Fig. 7F). Afterward, we measured the concentration of lipids in these two cell types. Our study showed that increased PTGER4 resulted in different degrees of reduction in cholesterol, triglycerides and free fatty acids (Fig. 7G). We also measured the senescence marker β-galactosidase in these two cell lines. indicated that elevated expression of PTGER4 facilitated the process of cellular senescence in renal cancer cells (Fig. 7H). The cell cycle flow results of the two cell lines also confirmed this phenomenon. The renal cancer cells in the group with high PTGER4 were mostly in the G0/G1 phase, while the low PTGER4 were mostly in the S/G2 phase (Fig. 7I). To acquire a deeper valuing of the relationship between PTGER4 and the proliferation and senescence of RCC cells, we extracted the proteins of the PTGER4 high-expression group and treatment group for western blot experiments of genes that are markers for the cell cycle and genes that are markers for senescence. The results indicated that the upregulation of PTGER4 expression caused a noteworthy decline the marker proteins` expression associated with the cell cycle, such as cyclinD1, cyclinD3, CDK4 and CDK6, while the levels of senescence-associated proteins, namely, P16, P21 and P53, exhibited a substantial augmentation (Fig. 7J).

### PTGER4 inhibited RCC progression in vivo

**Fig. 8:**
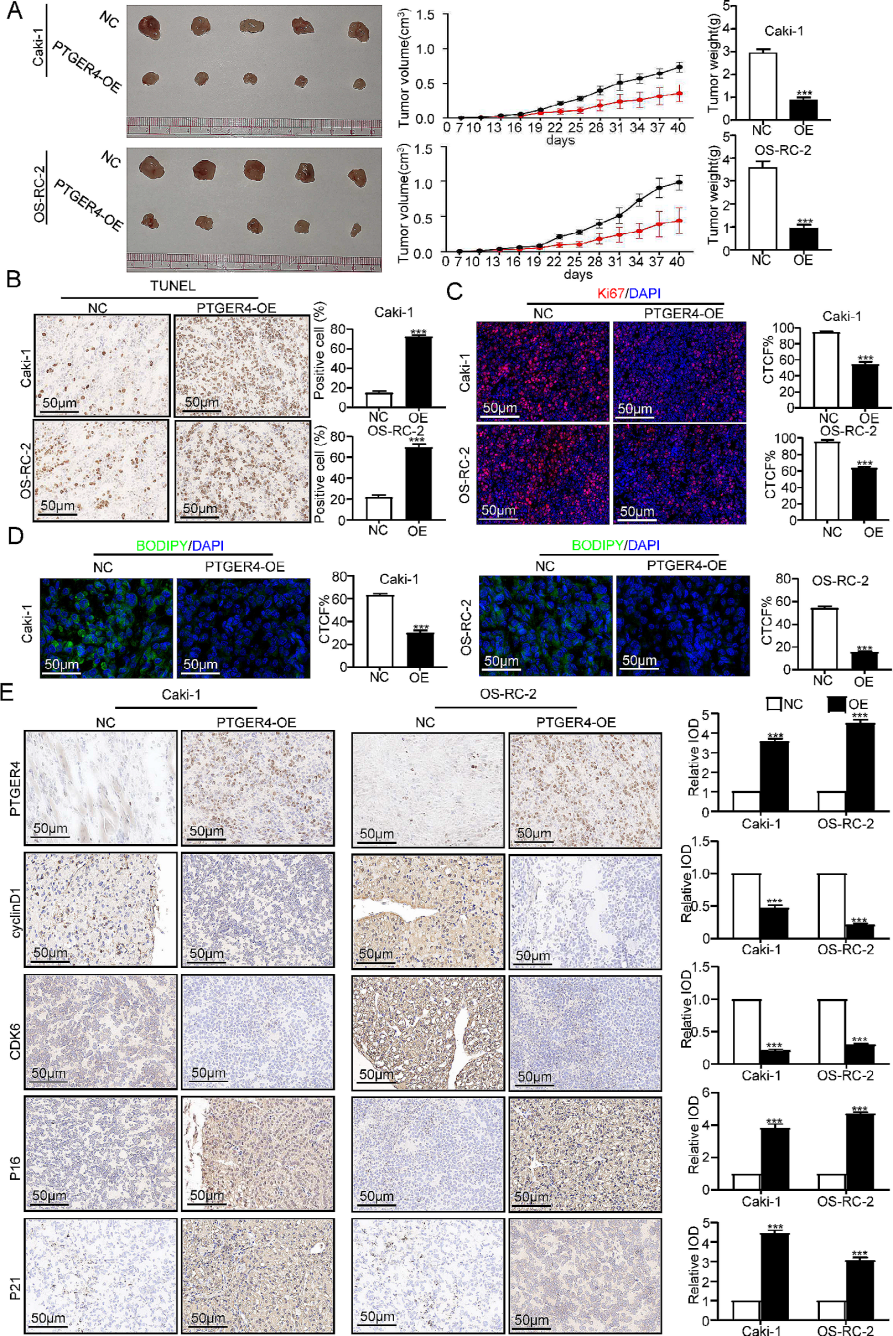
PTGER4 inhibited RCC progression in vivo. (**A**) The growth rate and weight of subcutaneous tumors after PTGER4 overexpression are shown. (**B**) The apoptosis of subcutaneous tumor cells in the PTGER4 overexpression group and control group is shown. (**C**) The proliferation of subcutaneous tumor cells in the PTGER4 overexpression group and control group is shown. (**D**) Neutral fatty acid levels in subcutaneous tumors of the PTGER4 overexpression group and control group are shown. (**E**) The expression of cell cycle-related proteins and cell senescence-related proteins in subcutaneous tumors of the PTGER4 overexpression group and control group is shown

To gain a deeper valuing of the effect of PTGER4 expression on renal cancer cells, we injected the PTGER4 overexpression lentivirus treatment group and the untreated group into 5-week-old C57 mice, with 5 mice in each group, and each injected cell volume was 1 × 107. From the seventh day after injection, the size of tumors located beneath the skin in mice was measured every three days. The results revealed that the PTGER4 overexpression group exhibited a reduced rate of tumor growth beneath the skin compared with the untreated. Additionally, the PTGER4 overexpression group exhibited a smaller weight in comparison to those in the untreated group (Fig. 8A). Subsequently, we performed section staining on the tumors of the four groups of mice. The findings revealed that the fluorescence concentration of TUNEL staining in the PTGER4 overexpression group was meaningfully superior to that in the untreated group (Fig. 8B), and the cell proliferation marker Ki67 staining results showed that the increase in PTGER4 expression caused a notable decline in Ki67 (Fig. 8C). At the same time, the findings from Bodipy staining revealed that the presence of neutral lipid droplets within the tumor exhibited varying degrees of reduction subsequent to the upregulation of PTGER4 expression (Fig. 8D). We also stained the sections for cell cycle and cell senescence-related markers. The findings indicated a notable decrease in the fluorescence intensity of cell cycle-related markers in the PTGER4 overexpression group compared to the untreated group. Conversely, the cell senescence-related markers exhibited the opposite trend (Fig. 8E). From the above experimental results, it can be inferred that the expression level of PTGER4 has a noteworthy effect on the overall growth activity of RCC cells both in vivo and in vitro. Furthermore, PTGER4 is closely associated with alterations in the cell cycle and the stimulation of senescence in renal cancer cells.

## Discussion

Metabolic reprogramming is a significant characteristic of neoplastic growth, plays an imperative function in the instigation and the advancement of tumors [25]. Based on its unique histological phenotype of cytoplasmic lipid deposition, renal cell carcinoma, especially ccRCC, is described as a metabolic disease [5, 26]. Most patients with RCC have a long overall survival time after surgery; the prognosis for advanced kidney cancer is extremely poor, despite the emergence of new targeted therapies, immunotherapy and combination therapy [27]. Senescent cells have been proposed as one of the emerging hallmarks and enabling features of cancer. Cellular senescence is a frequently observed phenomenon characterized by a state of irretrievable growth arrest in cells, characterized by alterations in cell structure and function, as well as the induction of a senescence-associated secretory phenotype (SASP) [28, 29]. The phenomenon of transitory senescence has been extensively studied in instances of therapy resistance, where it serves as a state of dormancy that evades the effects of therapeutic interventions on actively dividing cancer cells. However, it is plausible that transitory senescence plays a more widespread role in various stages of tumor formation, malignant advancement, and the expansion of cancer cells to distant sites [30]. Increasing evidence suggests that unraveling the mystery of cell senescence and metabolic reprogramming is expected to offer novel strategies for tumor treatment. During our research, we recognized the key genes interrelated to cell senescence and metabolism and constructed and validated an aging- and metabolism-related risk model for expecting the OS of people with ccRCC, which showed high accuracy in predicting overall survival and guiding clinical treatment measures.

In this research, we initially detected and examined the cell senescence- and metabolism-related DEGs in TCGA-KIRC, which were mainly enriched in lipid metabolism pathways. It is worth noting that these DEGs are also enriched in material transport, ligand‒receptor system and cell cycle-related pathways. RCC is a metabolic disease [5, 7]. These studies revealed that the metabolic adjustments in ccRCC were related not only to lipid metabolism but also to amino acid metabolism, the immune receptor response and cell cycle changes. Using the DEGs, a cluster analysis was conducted to identify two distinct subgroups among the patients. The analysis revealed noteworthy differences in OS of two groups. These findings suggest heterogeneity in the senescence and metabolism of renal cell carcinoma. Furthermore, patients with distinct patterns of aging metabolism exhibit varying prognoses. Following the construction of a PPI network, as well as the implementation of univariate and multivariate Cox, and so as the regression analysis of LASSO-Cox on the DEGs, we successfully identified seven significant mDEGs that are closely associated with survival, including NME2 (nucleoside diphosphate kinase 2), CD44 (CD44 molecule), ENO2 (enolase 2), COL1A1 (collagen type I alpha 1 chain), ENO1 (enolase 1), FGF1 (fibroblast growth factor 1), and PTGER4 (prostaglandin E receptor 4). These genes have previously been documented to take part in metabolic processes, aging mechanisms, or the advancement of tumors.

Previous studies have reported that overexpression of NME2 repressed metastasis of human oral squamous cell carcinoma, breast cancer and mouse melanoma cells [31–33]. However, the function of NME2 in ccRCC is still uncertain. Our study discovered a notable decline of NME2 expression in ccRCC tissues. This finding suggests that, in ccRCC, NME2 may behave as a cancer suppressor gene. It is well known that CD44 is a molecular marker for cancer stem cells (CSCs) [34]. Numerous studies have provided evidence indicating that CD44 is implicated in the alteration of various molecules and pathways, containing those associated with epithelial-mesenchymal transition (EMT) and matrix metalloproteinases (MMPs) [35], ring-related proteins [36], genes related to glycolytic enzymes [37], the Wnt/β-catenin pathway and the PI3K-Akt pathway [36]. These molecules and pathways play a crucial function in regulating proliferation, metastasis, and cancer cell mechanisms of resistance to drugs. In ccRCC, Chen et al. validated that CD44 promoted the advancement of ccRCC [38], and this finding aligns with the outcomes of our study. COL1A1 is the main constituent of type I collagen, and abnormal COL1A1 expression is associated with many cancers [39–41]. ENO2 (enolase 2) is a critical glycolytic enzyme in cancer metabolic processes [42]. Recent investigations have discovered that the overexpression of ENO2 encourages cell proliferation and glycolytic enrichment in papillary RCC [43]. FGF1, as a secretory regulator, can produce β mitogenic effects on cells from a variety of tissue sources [44–46]. In type I diabetic rat models, administration of FGF1 reduced lipolysis and hepatic glucose production [47]. Zhang et al. have provided evidence indicating that the absence of FGF1 expression is coupled with poor prediction in ccRCC patients [48]. PTGER4, the EP4 receptor of prostaglandin E2 (PGE2), is emerging as the most multifaceted and auspicious among the various PGE2 receptors. In the past, the EP4 receptor was shown to have anti-inflammatory, antithrombotic and vascular protective effects. The activation of the EP4 receptor and subsequent binding of the transcription factor CREB to the promoter region of the chemokine receptor CCR7 leads to a rise in receptor expression and enhances the migration of cells [49]. The EP4 receptor is primarily responsible for the anti-inflammatory properties of PGE2 [50], and it is widely recognized as a significant molecule that promotes tumor growth [51]. Wu et al. discerned that the activation of EP4 receptors causes the activation of RAP small GTPases through the cAMP/EPAC signaling pathway, leading to enhanced migration of renal cancer cells [52]. As the key gene in SeMRM, to gain a deeper valuing of the effect of PTGER4 in ccRCC, we performed a string of trials and verified the close correlation between PTGER4 and metabolism and cell senescence in ccRCC cells.

Using the information from these seven genes, SeMRS was computed and subsequently utilized to construct SeMRM. This model serves as a predictive instrument to define the prognosis of ccRCC patients. The TCGA-KIRC database serves as the primary dataset for training purposes, while two separate databases, namely, GSE29609 and ICGC-KIRC queues, are utilized as independent validation sets. Additional examination of the training and validation datasets revealed a strong correlation between SeMRS and the clinical characteristics of renal cell carcinoma, particularly the TNM stage and clinical grade. This suggests that the advancement of renal cell carcinoma is escorted by alterations in senescence and metabolism. The results of the varied analysis and univariate plus multivariate Cox regression analysis demonstrated that SeMRS served as a dependable and quantifiable predictive element, operating independently. It is important to highlight that SeMRS demonstrates superior accuracy in prognosticating tumor outcomes when compared to commonly utilized prognostic indicators presently in use (such as TNM staging and clinical grading). These findings indicate that the use of SeMRS shows potential as a clinical prognostic indicator for ccRCC. We measured the protein levels of CD44, ENO1, ENO2, FGF1, COL1A1, PTGER4 and NME2 in clinical samples obtained in the real world by IHC and calculated the SeMRS of each specimen using the constructed risk model formula for the targeting of discovering the prospect of clinical application of SeMRM,. The clinical information of patients collected from the real world was associated with SeMRS, and it was found that SeMRS had a noteworthy association with clinical and TNM stage, which was in accordance with the anticipated results. Simultaneously, it is noteworthy that the groups of individuals used for training and validation predominantly comprised individuals of non-Asian descent, while the clinical samples utilized in this investigation were exclusively sourced from China. This observation suggests that the SeMRM exhibits a robust level of generalizability. Thus, there is a scarcity of patient transcriptome data in the real world, and no further model validation has been performed. In the future, we will perform transcriptome sequencing on the collected samples to further validate the effectiveness and universality of SeMRM.

Recent studies have found that there is a correlation between cell metabolic reprogramming and TME remodeling [53–55]. ccRCC is a common and fatal urinary tract cancer. After treatment, approximately 50% of ccRCC patients will still have metastasis, and less than 10% of them can survive for 5 years. Targeted therapies such as tyrosine kinase inhibitors, which are now mainstream, are only palliative [56]. In our study, a computational risk scoring model based on SeMRM was used to quantify the level of tumor aging metabolism and comprehensively revealed the correlation between aging metabolic changes and the immune microenvironment to guide treatment in two different situations. In the high-SeMRS, there was a superior presence of T cells, some subtypes of B cells, and infiltration of macrophages. Simultaneously, the majority of traditional immune checkpoints, such as TNFRSF18, are also upregulated, which might inhibit the clearance of tumor cells by these cytotoxic cells. In addition, in comparison to other characteristics for clinical use, such as clinical and TNM stage, the characteristics of SeMRS can more effectively predict the tumor immune response of ccRCC patients. Therefore, quantifying the level of tumor aging metabolism by SeMRS has the potential to forecast the immune reaction of tumors and circumvent the need for immunosuppressive treatment in patients who exhibit a lack of immune response to reduce overtreatment.

Tumor metabolic reprogramming and cell senescence are part of the specific changes in tumors, and these changes have garnered increasing interest. RCC is essentially a metabolic disease characterized by a reprogramming of energy metabolism, a change that meets its needs for rapid growth and survival [57–60]. Under normal conditions, cells obtain energy through oxidative phosphorylation, an efficient energy-producing process. However, in RCC cells, due to impaired mitochondrial function or insufficient oxygen supply, RCC cells fulfill their needs for rapid growth and survival through glycolysis. By regulating the metabolic flux distribution of glycolysis, the energy production and growth of RCC cells can be influenced, based on which more effective therapeutic strategies may be developed in the future [61–63]. RCC is closely related to the function of mitochondria. Mitochondria, as the cell’s sapoenergetic region, produce ATP mainly through the process of oxidative phosphorylation to provide the cell with the required energy. However, during the development of RCC, mitochondrial bioenergy production and oxidative phosphorylation are often impaired, resulting in insufficient ATP production. To compensate for this energy gap, the level of glycolysis is elevated in RCC cells. Mitochondrial function is closely linked to lipid metabolism and is involved in fatty acid oxidation and ketone body production. In RCC cells, fatty acid oxidation is inhibited due to impaired mitochondrial function, which may lead to intracellular lipid accumulation. This change further exacerbates the metabolic abnormalities and malignant phenotype of RCC cells [61, 64–66]. We identified the significant mDEG, demonstrated the landscape of senescence and metabolism, and formed a SeMRM to conduct a methodical examination of the correlation between the alteration of tumor metabolism, the recurrence of tumors, cellular senescence changes, and therapeutic responses in renal cell carcinoma. Modern medical treatment for advanced ccRCC patients has a variety of treatment methods, but according to the gene expression of patients, selecting the corresponding targeted drugs for treatment has become a recognized means of diagnosis and treatment. In the present study, at least 12 genes that accompany the pathogenesis of RCC were shown to participate in basic metabolic processes. RCC is increasingly considered a cell metabolic disease. At the same time, aging is becoming increasingly intricately linked to the occurrence of ccRCC [67, 68]. Chemotherapy resistance is toughly networked to drug metabolism. In our study, we found that patients in diverse risk groups had diverse sensitivities to different molecular targeted drugs; for example, the low-SeMRS group exhibited a heightened level of sensitivity toward the first-line targeted drugs sorafenib and pazopanib. As cancer molecular targeted therapy becomes more popular, we have also made predictions about the responsiveness of various subgroups to other commonly used molecular targeted drugs. This will help guide patients with different conditions due to senescence and metabolism to choose the most suitable targeted drugs with greater accuracy. These findings provide capacity for the future application of SeMRM in clinical directions.

In summary, our research identified the significant mDEG, exhibited the overall senescent and metabolic landscape of ccRCC, and structured a SeMRM. SeMRS demonstrated excellent diagnostic accuracy in forecasting OS and treatment response in patients with ccRCC. Simultaneously, PTGER4 was probed both in vitro and vivo in the experimental mode, and the impact of its expression level on lipid metabolism and the cell cycle of renal cancer cells was found. In the future, we anticipate that additional clinical studies will confirm the practicality of the constructed SeMRM.

### Electronic supplementary material

Below is the link to the electronic supplementary material.

Supplementary Material 1

## Data Availability

The datasets used and/or analyzed during the current study are available from the corresponding author on reasonable request.

## References

[1] Siegel RL, Miller KD, Wagle NS, Jemal A (2023). Cancer statistics, 2023. CA Cancer J Clin.

[2] Pontes O, Oliveira-Pinto S, Baltazar F, Costa M (2022). Renal cell carcinoma therapy: current and new drug candidates. Drug Discov Today.

[3] Rosellini M, Marchetti A, Mollica V, Rizzo A, Santoni M, Massari F (2023). Prognostic and predictive biomarkers for immunotherapy in advanced renal cell carcinoma. Nat Rev Urol.

[4] Rini BI, Plimack ER, Stus V, Gafanov R, Hawkins R, Nosov D, Pouliot F, Alekseev B, Soulières D, Melichar B (2019). Pembrolizumab plus Axitinib versus Sunitinib for Advanced Renal-Cell Carcinoma. N Engl J Med.

[5] Wettersten HI, Aboud OA, Lara PN, Weiss RH (2017). Metabolic reprogramming in clear cell renal cell carcinoma. Nat Rev Nephrol.

[6] Jonasch E, Walker CL, Rathmell WK (2021). Clear cell renal cell carcinoma ontogeny and mechanisms of lethality. Nat Rev Nephrol.

[7] Zhou L, Luo Y, Liu Y, Zeng Y, Tong J, Li M, Hou Y, Du K, Qi Y, Pan W (2023). Fatty acid oxidation mediated by Malonyl-CoA decarboxylase represses renal cell carcinoma progression. Cancer Res.

[8] Cassim S, Pouyssegur J. Tumor Microenvironment: a metabolic player that shapes the Immune response. Int J Mol Sci 2019, 21(1). 10.3390/ijms21010157 PMC6982275 31881671

[9] Liu G, Parant JM, Lang G, Chau P, Chavez-Reyes A, El-Naggar AK, Multani A, Chang S, Lozano G (2004). Chromosome stability, in the absence of apoptosis, is critical for suppression of tumorigenesis in Trp53 mutant mice. Nat Genet.

[10] Rakesh R, PriyaDharshini LC, Sakthivel KM, Rasmi RR (2022). Role and regulation of autophagy in cancer. Biochim Biophys Acta Mol Basis Dis.

[11] Domen A, Deben C, Verswyvel J, Flieswasser T, Prenen H, Peeters M, Lardon F, Wouters A (2022). Cellular senescence in cancer: clinical detection and prognostic implications. J Exp Clin Cancer Res.

[12] Roger L, Tomas F, Gire V. Mechanisms and regulation of Cellular Senescence. Int J Mol Sci 2021, 22(23). 10.3390/ijms222313173 PMC8658264 34884978

[13] Kawakami I, Yoshino H, Fukumoto W, Tamai M, Okamura S, Osako Y, Sakaguchi T, Inoguchi S, Matsushita R, Yamada Y (2022). Targeting of the glutamine transporter SLC1A5 induces cellular senescence in clear cell renal cell carcinoma. Biochem Biophys Res Commun.

[14] Gerhauser C, Favero F, Risch T, Simon R, Feuerbach L, Assenov Y, Heckmann D, Sidiropoulos N, Waszak SM, Hübschmann D (2018). Molecular evolution of early-onset prostate Cancer identifies molecular risk markers and clinical trajectories. Cancer Cell.

[15] Taylor BS, Schultz N, Hieronymus H, Gopalan A, Xiao Y, Carver BS, Arora VK, Kaushik P, Cerami E, Reva B (2010). Integrative genomic profiling of human prostate cancer. Cancer Cell.

[16] Shi T, Ma Y, Yu L, Jiang J, Shen S, Hou Y, Wang T. Cancer Immunotherapy: a focus on the regulation of Immune checkpoints. Int J Mol Sci 2018, 19(5). 10.3390/ijms19051389 PMC5983802 29735917

[17] Ritchie ME, Phipson B, Wu D, Hu Y, Law CW, Shi W, Smyth GK (2015). Limma powers differential expression analyses for RNA-sequencing and microarray studies. Nucleic Acids Res.

[18] Yu G, Wang LG, Han Y, He QY (2012). clusterProfiler: an R package for comparing biological themes among gene clusters. Omics.

[19] Wilkerson MD, Hayes DN (2010). ConsensusClusterPlus: a class discovery tool with confidence assessments and item tracking. Bioinformatics.

[20] Chin CH, Chen SH, Wu HH, Ho CW, Ko MT, Lin CY (2014). cytoHubba: identifying hub objects and sub-networks from complex interactome. BMC Syst Biol.

[21] Engebretsen S, Bohlin J (2019). Statistical predictions with glmnet. Clin Epigenetics.

[22] Mayakonda A, Lin DC, Assenov Y, Plass C, Koeffler HP (2018). Maftools: efficient and comprehensive analysis of somatic variants in cancer. Genome Res.

[23] Maeser D, Gruener RF, Huang RS. oncoPredict: an R package for predicting in vivo or cancer patient drug response and biomarkers from cell line screening data. Brief Bioinform 2021, 22(6). 10.1093/bib/bbab260 PMC8574972 34260682

[24] Zhou L, Zhang C, Yang X, Liu L, Hu J, Hou Y, Tao H, Sugimura H, Chen Z, Wang L (2021). Melatonin inhibits lipid accumulation to repress prostate cancer progression by mediating the epigenetic modification of CES1. Clin Transl Med.

[25] Miranda-Gonçalves V, Lameirinhas A, Henrique R, Baltazar F, Jerónimo C (2020). The metabolic landscape of urological cancers: new therapeutic perspectives. Cancer Lett.

[26] Linehan WM, Schmidt LS, Crooks DR, Wei D, Srinivasan R, Lang M, Ricketts CJ (2019). The metabolic basis of kidney Cancer. Cancer Discov.

[27] Choueiri TK, Motzer RJ (2017). Systemic therapy for metastatic renal-cell carcinoma. N Engl J Med.

[28] Coppé JP, Patil CK, Rodier F, Sun Y, Muñoz DP, Goldstein J, Nelson PS, Desprez PY, Campisi J (2008). Senescence-associated secretory phenotypes reveal cell-nonautonomous functions of oncogenic RAS and the p53 tumor suppressor. PLoS Biol.

[29] Coppé JP, Desprez PY, Krtolica A, Campisi J (2010). The senescence-associated secretory phenotype: the dark side of tumor suppression. Annu Rev Pathol.

[30] Wang Z, Dabrosin C, Yin X, Fuster MM, Arreola A, Rathmell WK, Generali D, Nagaraju GP, El-Rayes B, Ribatti D (2015). Broad targeting of angiogenesis for cancer prevention and therapy. Semin Cancer Biol.

[31] Lo Muzio L, Mignogna MD, Pannone G, Staibano S, Procaccini M, Serpico R, De Rosa G, Scully C (1999). The NM23 gene and its expression in oral squamous cell carcinoma. Oncol Rep.

[32] Baba H, Urano T, Okada K, Furukawa K, Nakayama E, Tanaka H, Iwasaki K, Shiku H (1995). Two isotypes of murine nm23/nucleoside diphosphate kinase, nm23-M1 and nm23-M2, are involved in metastatic suppression of a murine melanoma line. Cancer Res.

[33] Russell RL, Pedersen AN, Kantor J, Geisinger K, Long R, Zbieranski N, Townsend A, Shelton B, Brünner N, Kute TE (1998). Relationship of nm23 to proteolytic factors, proliferation and motility in breast cancer tissues and cell lines. Br J Cancer.

[34] Yin T, Wang G, He S, Liu Q, Sun J, Wang Y (2016). Human cancer cells with stem cell-like phenotype exhibit enhanced sensitivity to the cytotoxicity of IL-2 and IL-15 activated natural killer cells. Cell Immunol.

[35] Zhang Y, Thant AA, Machida K, Ichigotani Y, Naito Y, Hiraiwa Y, Senga T, Sohara Y, Matsuda S, Hamaguchi M (2002). Hyaluronan-CD44s signaling regulates matrix metalloproteinase-2 secretion in a human lung carcinoma cell line QG90. Cancer Res.

[36] Chang G, Zhang H, Wang J, Zhang Y, Xu H, Wang C, Zhang H, Ma L, Li Q, Pang T (2013). CD44 targets Wnt/β-catenin pathway to mediate the proliferation of K562 cells. Cancer Cell Int.

[37] Miletti-González KE, Murphy K, Kumaran MN, Ravindranath AK, Wernyj RP, Kaur S, Miles GD, Lim E, Chan R, Chekmareva M (2012). Identification of function for CD44 intracytoplasmic domain (CD44-ICD): modulation of matrix metalloproteinase 9 (MMP-9) transcription via novel promoter response element. J Biol Chem.

[38] Chen Z, Zheng Z, Xie Y, Zhong Q, Shangguan W, Zhang Y, Zhu D, Xie W (2021). Circular RNA circPPP6R3 upregulates CD44 to promote the progression of clear cell renal cell carcinoma via sponging miR-1238-3p. Cell Death Dis.

[39] Wu W, Yang Z, Long F, Luo L, Deng Q, Wu J, Ouyang S, Tang D (2020). COL1A1 and MZB1 as the hub genes influenced the proliferation, invasion, migration and apoptosis of rectum adenocarcinoma cells by weighted correlation network analysis. Bioorg Chem.

[40] Ma HP, Chang HL, Bamodu OA, Yadav VK, Huang TY, Wu ATH, Yeh CT, Tsai SH, Lee WH. Collagen 1A1 (COL1A1) is a Reliable Biomarker and putative therapeutic target for Hepatocellular Carcinogenesis and Metastasis. Cancers (Basel) 2019, 11(6). 10.3390/cancers11060786 PMC6627889 31181620

[41] Li Y, Sun R, Zhao X, Sun B (2021). RUNX2 promotes malignant progression in gastric cancer by regulating COL1A1. Cancer Biomark.

[42] Gao L, Yang F, Tang D, Xu Z, Tang Y, Yang D, Sun D, Chen Z, Teng Y (2023). Mediation of PKM2-dependent glycolytic and non-glycolytic pathways by ENO2 in head and neck cancer development. J Exp Clin Cancer Res.

[43] Huebbers CU, Adam AC, Preuss SF, Schiffer T, Schilder S, Guntinas-Lichius O, Schmidt M, Klussmann JP, Wiesner RJ (2015). High glucose uptake unexpectedly is accompanied by high levels of the mitochondrial ß-F1-ATPase subunit in head and neck squamous cell carcinoma. Oncotarget.

[44] Kan M, Huang JS, Mansson PE, Yasumitsu H, Carr B, McKeehan WL (1989). Heparin-binding growth factor type 1 (acidic fibroblast growth factor): a potential biphasic autocrine and paracrine regulator of hepatocyte regeneration. Proc Natl Acad Sci U S A.

[45] Wiedłocha A, Falnes PO, Rapak A, Muñoz R, Klingenberg O, Olsnes S (1996). Stimulation of proliferation of a human osteosarcoma cell line by exogenous acidic fibroblast growth factor requires both activation of receptor tyrosine kinase and growth factor internalization. Mol Cell Biol.

[46] Nabel EG, Yang ZY, Plautz G, Forough R, Zhan X, Haudenschild CC, Maciag T, Nabel GJ (1993). Recombinant fibroblast growth factor-1 promotes intimal hyperplasia and angiogenesis in arteries in vivo. Nature.

[47] Perry RJ, Lee S, Ma L, Zhang D, Schlessinger J, Shulman GI (2015). FGF1 and FGF19 reverse diabetes by suppression of the hypothalamic-pituitary-adrenal axis. Nat Commun.

[48] Zhang X, Wang Z, Zeng Z, Shen N, Wang B, Zhang Y, Shen H, Lu W, Wei R, Ma W (2021). Bioinformatic analysis identifying FGF1 gene as a new prognostic indicator in clear cell renal cell carcinoma. Cancer Cell Int.

[49] Côté SC, Pasvanis S, Bounou S, Dumais N (2009). CCR7-specific migration to CCL19 and CCL21 is induced by PGE(2) stimulation in human monocytes: involvement of EP(2)/EP(4) receptors activation. Mol Immunol.

[50] Tang EH, Libby P, Vanhoutte PM, Xu A (2012). Anti-inflammation therapy by activation of prostaglandin EP4 receptor in cardiovascular and other inflammatory diseases. J Cardiovasc Pharmacol.

[51] Chell SD, Witherden IR, Dobson RR, Moorghen M, Herman AA, Qualtrough D, Williams AC, Paraskeva C (2006). Increased EP4 receptor expression in colorectal cancer progression promotes cell growth and anchorage independence. Cancer Res.

[52] Wu J, Zhang Y, Frilot N, Kim JI, Kim WJ, Daaka Y (2011). Prostaglandin E2 regulates renal cell carcinoma invasion through the EP4 receptor-rap GTPase signal transduction pathway. J Biol Chem.

[53] Reina-Campos M, Moscat J, Diaz-Meco M (2017). Metabolism shapes the tumor microenvironment. Curr Opin Cell Biol.

[54] Ocaña MC, Martínez-Poveda B, Quesada AR, Medina M (2019). Metabolism within the tumor microenvironment and its implication on cancer progression: an ongoing therapeutic target. Med Res Rev.

[55] Zhou L, Du K, Dai Y, Zeng Y, Luo Y, Ren M, Pan W, Liu Y, Zhang L, Zhu R (2024). Metabolic reprogramming based on RNA sequencing of gemcitabine-resistant cells reveals the FASN gene as a therapeutic for bladder cancer. J Translational Med.

[56] Koul H, Huh JS, Rove KO, Crompton L, Koul S, Meacham RB, Kim FJ (2011). Molecular aspects of renal cell carcinoma: a review. Am J Cancer Res.

[57] di Meo NA, Lasorsa F, Rutigliano M, Milella M, Ferro M, Battaglia M, Ditonno P, Lucarelli G (2023). The dark side of lipid metabolism in prostate and renal carcinoma: novel insights into molecular diagnostic and biomarker discovery. Expert Rev Mol Diagn.

[58] Lucarelli G, Loizzo D, Franzin R, Battaglia S, Ferro M, Cantiello F, Castellano G, Bettocchi C, Ditonno P, Battaglia M (2019). Metabolomic insights into pathophysiological mechanisms and biomarker discovery in clear cell renal cell carcinoma. Expert Rev Mol Diagn.

[59] di Meo NA, Lasorsa F, Rutigliano M, Loizzo D, Ferro M, Stella A, Bizzoca C, Vincenti L, Pandolfo SD, Autorino R et al. Renal Cell Carcinoma as a metabolic disease: an update on main pathways, potential biomarkers, and therapeutic targets. Int J Mol Sci 2022, 23(22). 10.3390/ijms232214360 PMC9698586 36430837

[60] De Marco S, Torsello B, Minutiello E, Morabito I, Grasselli C, Bombelli S, Zucchini N, Lucarelli G, Strada G, Perego RA (2023). The cross-talk between Abl2 tyrosine kinase and TGFβ1 signalling modulates the invasion of clear cell renal cell carcinoma cells. FEBS Lett.

[61] Bianchi C, Meregalli C, Bombelli S, Di Stefano V, Salerno F, Torsello B, De Marco S, Bovo G, Cifola I, Mangano E (2017). The glucose and lipid metabolism reprogramming is grade-dependent in clear cell renal cell carcinoma primary cultures and is targetable to modulate cell viability and proliferation. Oncotarget.

[62] Ragone R, Sallustio F, Piccinonna S, Rutigliano M, Vanessa G, Palazzo S, Lucarelli G, Ditonno P, Battaglia M, Fanizzi FP et al. Renal cell carcinoma: a study through NMR-Based Metabolomics combined with transcriptomics. Dis (Basel Switzerland) 2016, 4(1). 10.3390/diseases4010007 PMC5456302 28933387

[63] Lucarelli G, Galleggiante V, Rutigliano M, Sanguedolce F, Cagiano S, Bufo P, Lastilla G, Maiorano E, Ribatti D, Giglio A (2015). Metabolomic profile of glycolysis and the pentose phosphate pathway identifies the central role of glucose-6-phosphate dehydrogenase in clear cell-renal cell carcinoma. Oncotarget.

[64] Lucarelli G, Rutigliano M, Sallustio F, Ribatti D, Giglio A, Lepore Signorile M, Grossi V, Sanese P, Napoli A, Maiorano E (2018). Integrated multi-omics characterization reveals a distinctive metabolic signature and the role of NDUFA4L2 in promoting angiogenesis, chemoresistance, and mitochondrial dysfunction in clear cell renal cell carcinoma. Aging.

[65] Bombelli S, Torsello B, De Marco S, Lucarelli G, Cifola I, Grasselli C, Strada G, Bovo G, Perego RA, Bianchi C (2020). 36-kDa annexin A3 isoform negatively modulates lipid storage in Clear Cell Renal Cell Carcinoma cells. Am J Pathol.

[66] Lucarelli G, Rutigliano M, Loizzo D, di Meo NA, Lasorsa F, Mastropasqua M, Maiorano E, Bizzoca C, Vincenti L, Battaglia M et al. MUC1 tissue expression and its soluble form CA15-3 identify a clear cell renal cell carcinoma with distinct metabolic Profile and Poor Clinical Outcome. Int J Mol Sci 2022, 23(22). 10.3390/ijms232213968 PMC9696833 36430448

[67] Linehan WM, Srinivasan R, Schmidt LS (2010). The genetic basis of kidney cancer: a metabolic disease. Nat Rev Urol.

[68] Linehan WM, Ricketts CJ (2013). The metabolic basis of kidney cancer. Semin Cancer Biol.

