# Supplementary material for: Cellular senescence and metabolic reprogramming model based on bulk/single-cell RNA sequencing reveals PTGER4 as a therapeutic target for ccRCC
Source: BMC Cancer. 2024 Apr 11;24:451. doi: 10.1186/s12885-024-12234-5 (PMC11007942; doi:10.1186/s12885-024-12234-5)
Supplement: Supplementary file 1 — Supplementary Material 1 [file 12885_2024_12234_MOESM1_ESM.pdf]

# Supplementary Figure 1

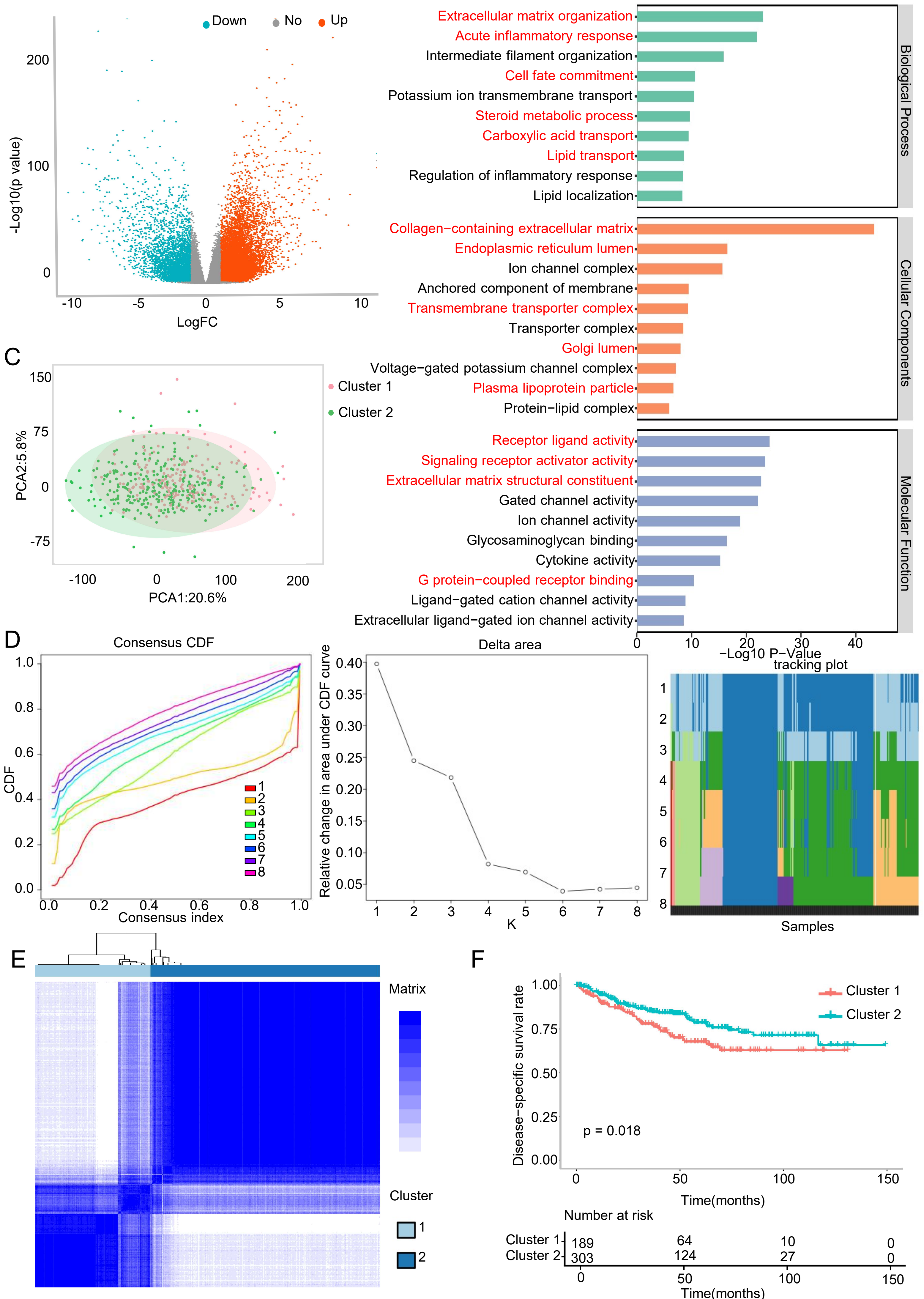

# Supplementary Figure 2

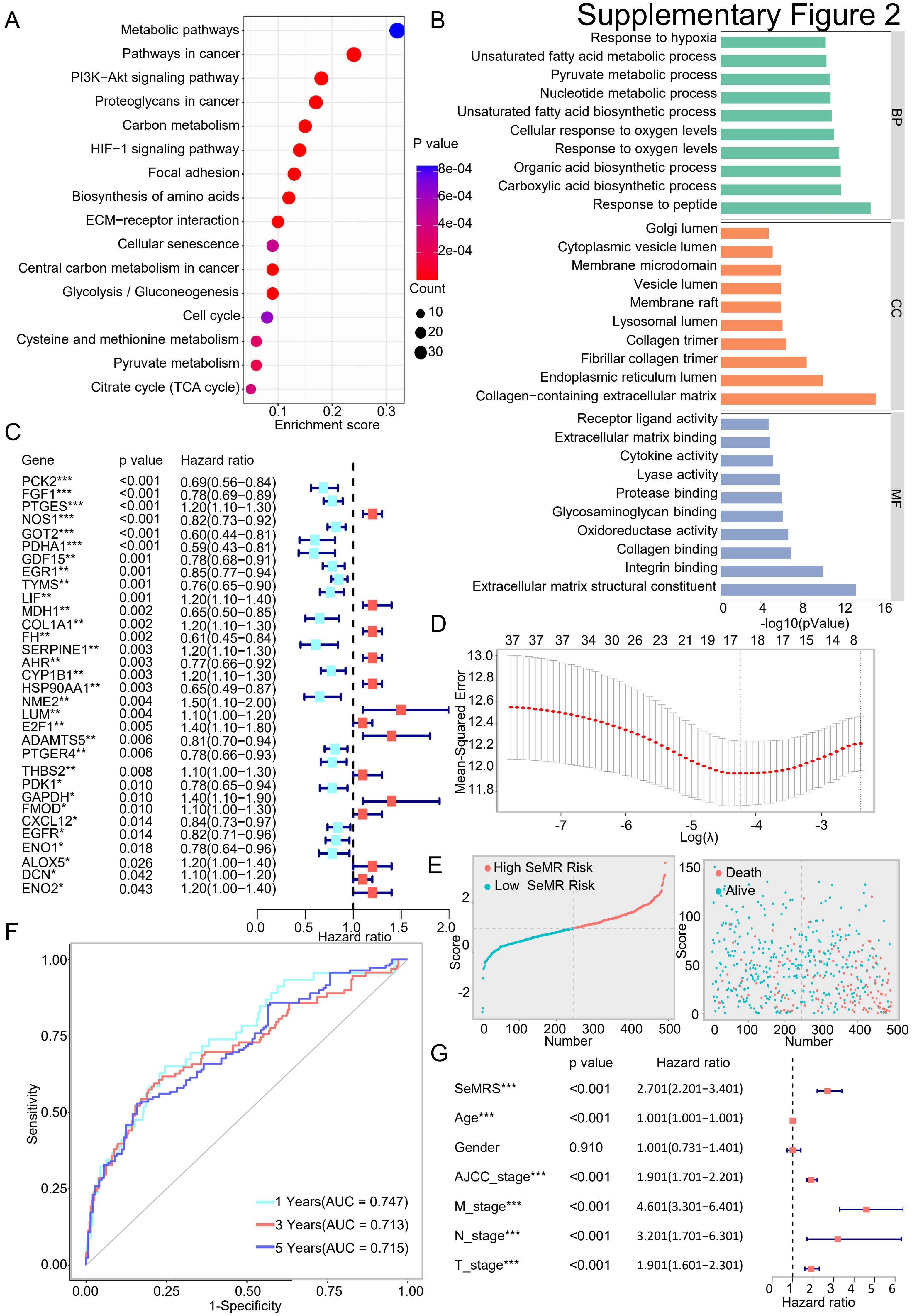

# Supplementary Figure 3

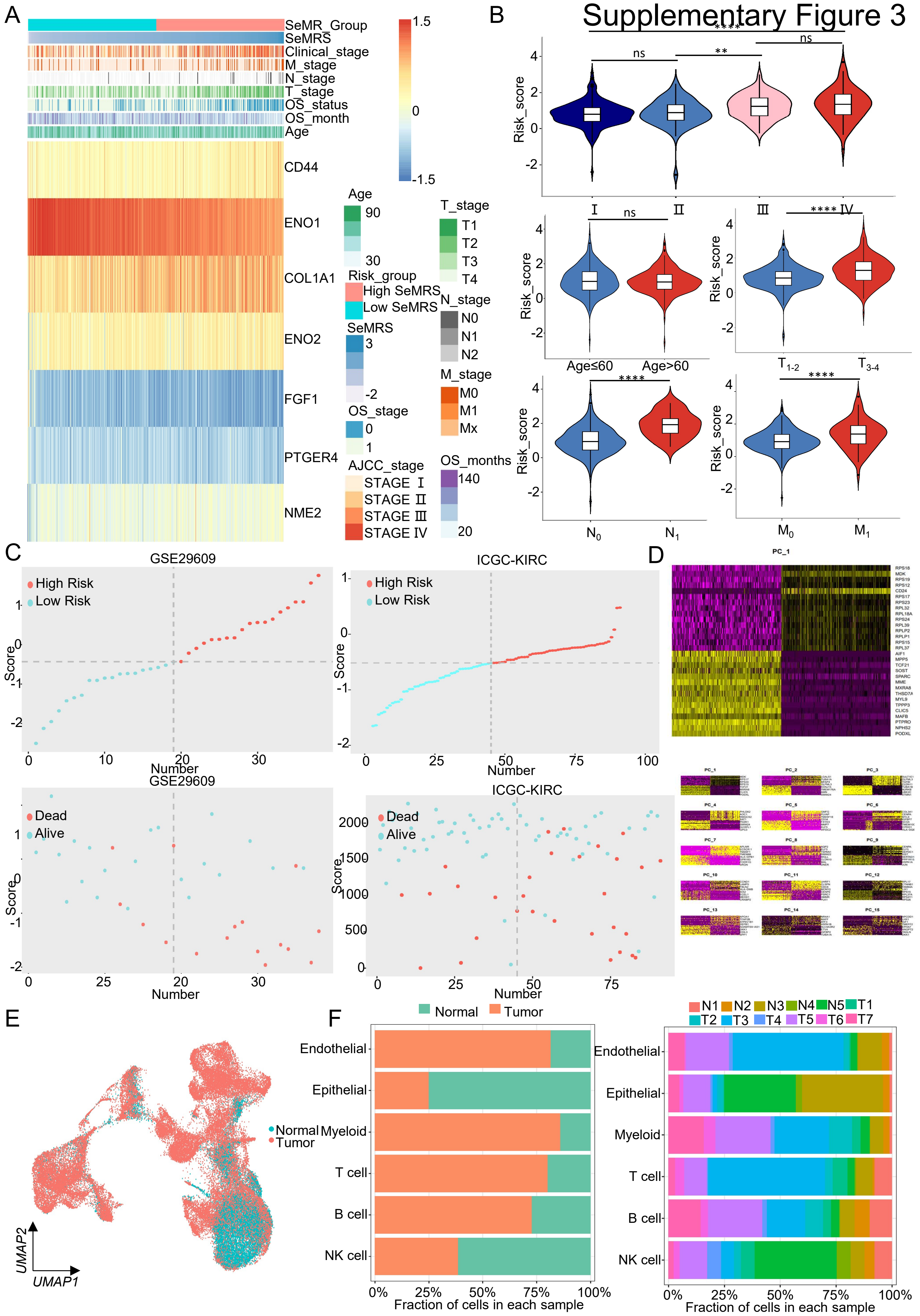

# Supplementary Figure 4

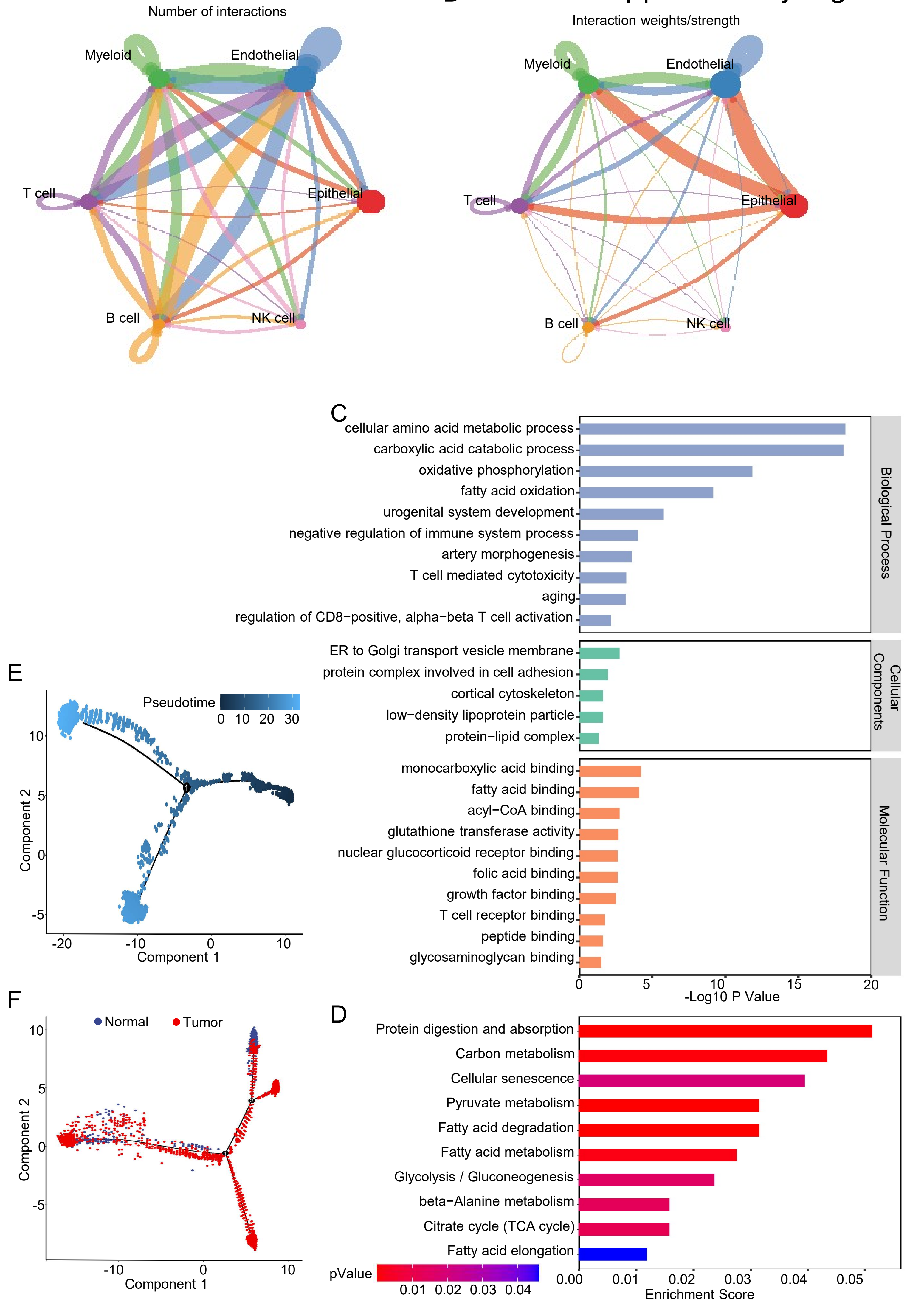

Supplementary Figure 5

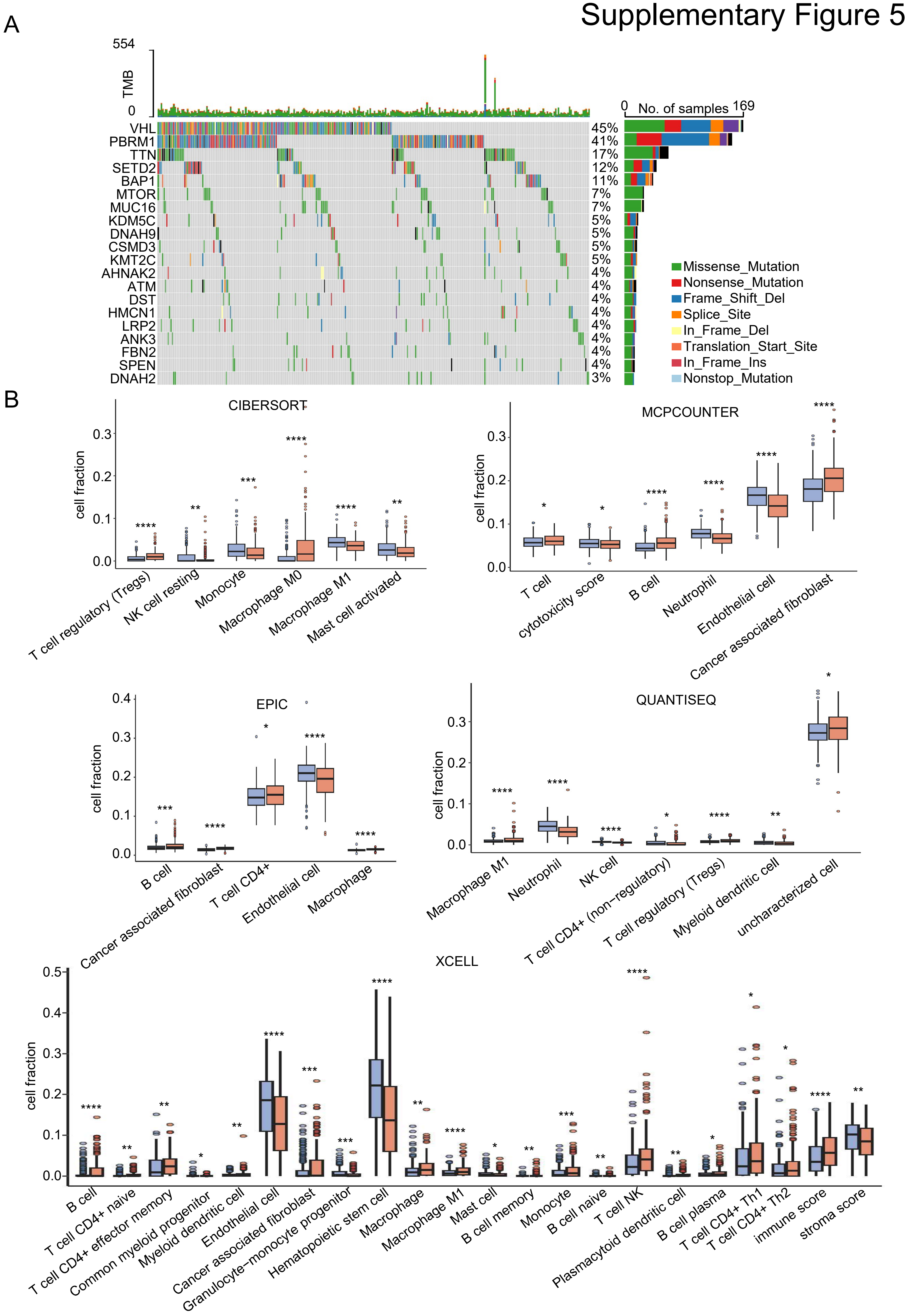

Supplementary Figure 6

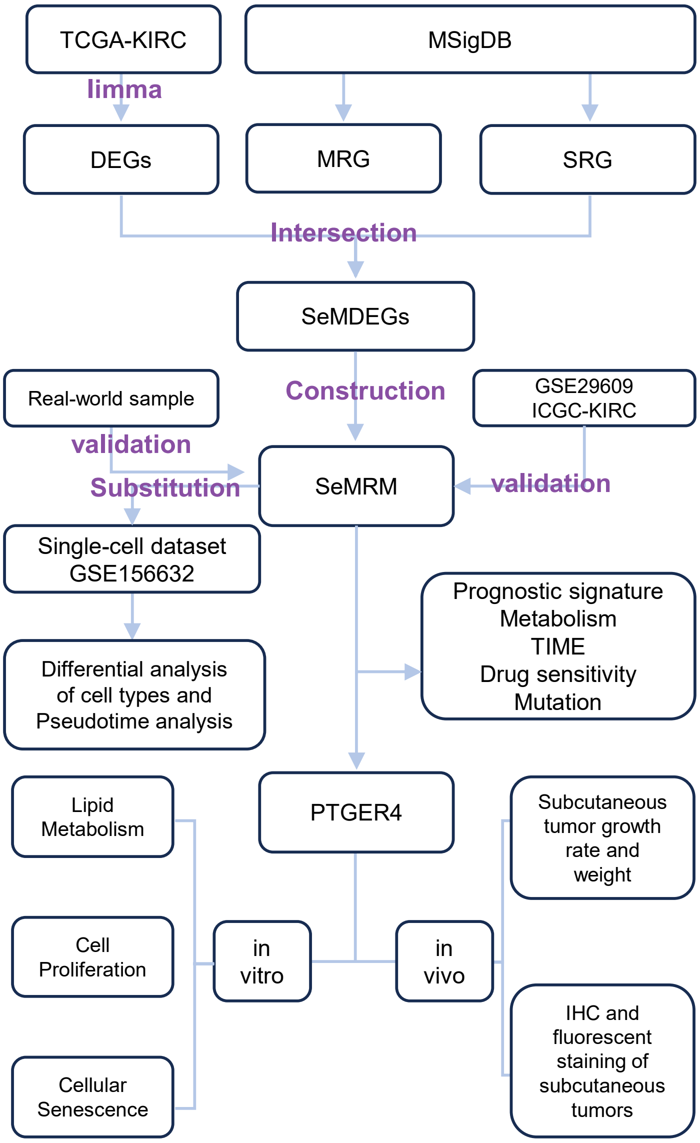

Supplementary Figure 7

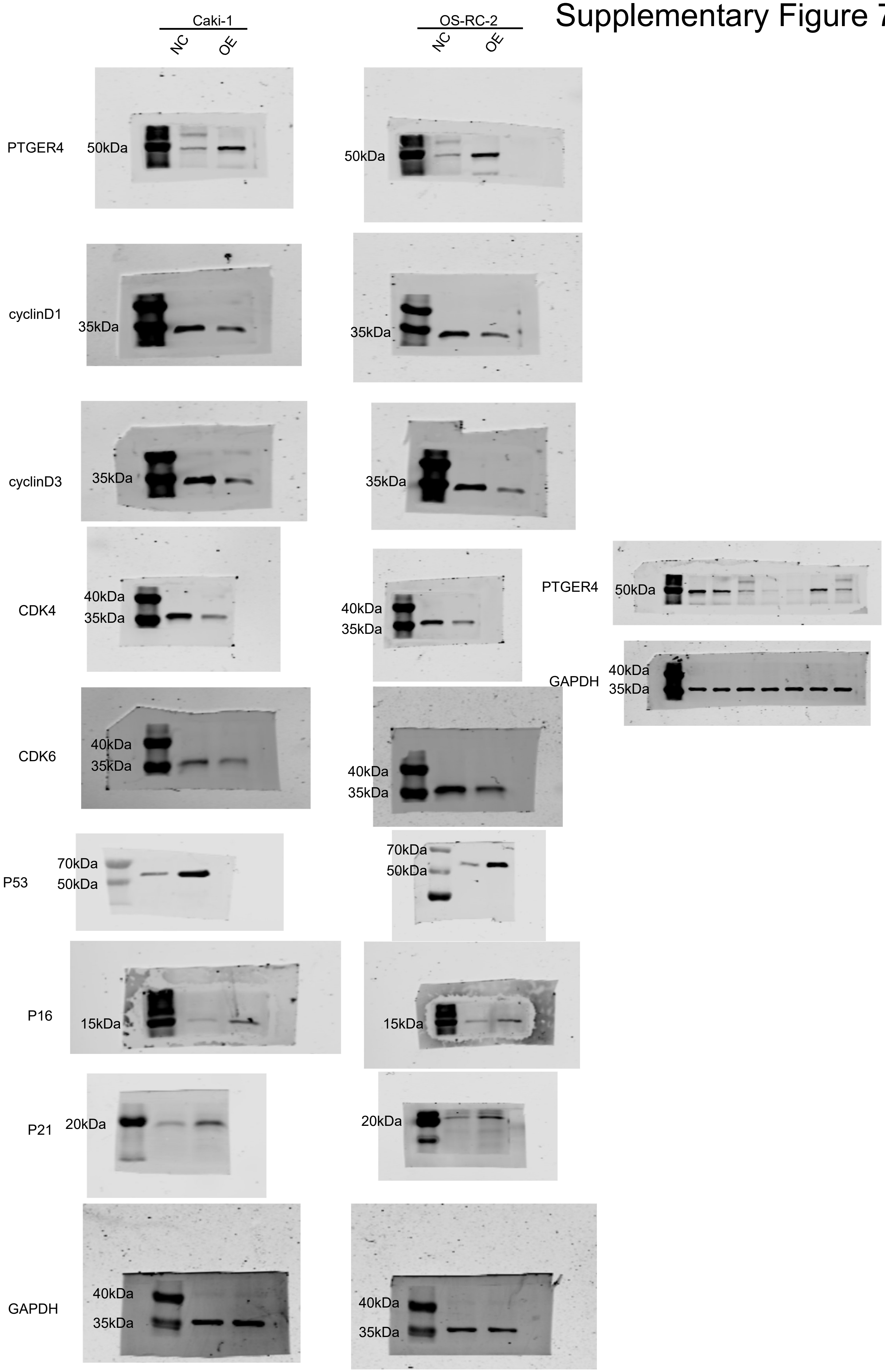

**Supplementary Figure Legends**

**Supplementary Figure 1 |** (**A**) Volcano maps of differentially expressed genes (DEGs) between ccRCC tissues and normal kidney tissues in The Cancer Genome Atlas (TCGA) KIRC database. Sky blue represents downregulated genes in ccRCC, and orange red represents upregulated genes in ccRCC. (**B**) Gene ontology (GO) enrichment analysis of mDEG. **(C)** Principal component analysis (PCA) of the TCGA KIRC database with the best k = 2. **(D)** Consensus clustering distribution function (CDF), area under the CDF curve increment, and k = 2 to 9 in the tracking plot. **(E)** Consistent clustering of TCGA-KIRC queues based on mDEG. Optimal k = 2 uniform matrix. **(F)** Kaplan–Meier analysis of overall survival (OS) curves of patients in different clusters.

**Supplementary Figure 2 |** (**A-B**) GO and KEGG analysis of these fundamental genes. Adjusted $p < 0.01$ and $p < 0.05$ were considered significant. (**C**) A forest map of OS-related key mDEGs based on univariate Cox regression analysis of the top 100 central genes, with $p < 0.05$ identifying the main OS-related mDEGs. (**D-E**) LASSO Cox regression of OS-related key senescence-metabolism-related differentially expressed genes (mDEG) and risk score distribution based on the median senescence-metabolism-related risk score (SeMRM) in the Cancer Genome Atlas (TCGA) KIRC. Blue represents the low-SeMRS subgroup, while red represents the high-SeMRS subgroup. The distribution of surviving patients (blue) or dead patients (red) in the subgroup. (**F**) Receiver operating characteristic (ROC) curves for predicting 1-year, 3-year and 5-year OS in the TCGA KIRC database. (**G**) Univariate Cox regression analysis of SeMRM and clinical features.

**Supplementary Figure 3 |** (**A**) The heatmap, SeMRM characteristics and clinical characteristics of the seven constituent genes of SeMRM in the TCGA-KIRC database. (**B**) SeMRS was positively interrelated with clinical grade and TNM stage but not with age. Student's t test; (**C**) The dispersal of SeMRS in Taylor and ICGC KIRC databases centered around the median SeMRS. Blue represents the low-SeMRS group, while red represents the high-SeMRS group and the distribution of living patients (blue) or dead patients (red) in subgroups. (**D**) Heatmap of differentially expressed genes among each single-cell data clustering subtype. (**E**) UMAP of cancer and cancer-adjacent samples

in single-cell data. (**F**) Bar chart of SeMRS sample distribution according to different sample sources.

**Supplementary Figure 4 |** (**A-B**) Cell communication between different cell subtypes. (**C-D**) Visualization of GO and KEGG results for the DEGs obtained from single-cell data using SeMRS as the clustering criterion. (**E**) Map of T-cell subtype pseudotime analysis. (**F**) Distribution map of T-cell subtypes based on sample source.

**Supplementary Figure 5 |** (**A**) Top 10 genes with the highest mutation rates in TCGA KIRC samples. Different colors represent diverse mutation types. The top shows the total number of mutations, while the right shows the percentage of mutations. (**B**) The proportions of TME cells in different SeMRS groups employed five independent processes (CIBERSORT, XCELL, QUANTISEQ, MCPCOUNTER and EPIC).

**Supplementary Figure 6 |** Flowchart of model construction and phenotypic validation in the study.

**Supplementary Figure 7 |** Original image of Western blot experiment on Figure 7J.
